# Fish 3D Clustering App (F3DCA): A Non-Supervised Clustering Method with a Machine Learning Autoencoder for Analyzing 3D Trajectories of Three Fish Species

**DOI:** 10.3390/biology15191768

**Published:** 2026-10-04

**Authors:** Ali Farhan, Gilbert Audira, Cao Thang Luong, Chih-Hsin Hung, Tzong-Rong Ger, Chung-Der Hsiao

**Affiliations:** 1 Department of Chemistry, Chung Yuan Christian University, Taoyuan 32023, Taiwan; smalifarhan@gmail.com (A.F.); gilbertaudira@yahoo.com (G.A.); 2 Department of Bioscience Technology, Chung Yuan Christian University, Taoyuan 32023, Taiwan; 3 Department of Chemical Engineering, Institute of Biotechnology and Chemical Engineering, I-Shou University, Da-Shu, Kaohsiung City 84001, Taiwan; thang.luongcao@gmail.com (C.T.L.); chhung@isu.edu.tw (C.-H.H.); 4 Department of Biomedical Engineering, National Yang Ming Chiao Tung University, Taipei 112304, Taiwan; sunbow@nycu.edu.tw; 5 Department of Biomedical Engineering, Chung Yuan Christian University, Taoyuan 32023, Taiwan; 6 Research Center for Aquatic Toxicology and Pharmacology, Chung Yuan Christian University, Taoyuan 32023, Taiwan

**Keywords:** clustering, auto-encoder, zebrafish, three-dimensional, trajectory

## Abstract

Fish behaviors are widely studied in various research fields due to their relevance to many aspects, including neurodegenerative processes, since they can impair motor control, complex behavior, habituation, and response to environmental stimuli. 3D locomotion trajectory analysis is one of the assays to analyze complex behaviors in fish. However, while existing 3D tracking software can capture the 3D trajectories of fish, they still rely heavily on manual or semi-automated analysis. Moreover, traditional analysis methods are inadequate for handling the high complexity of raw 3D data (X, Y, Z coordinates), and manual/semi-automated procedures introduce subjective human bias and are highly time-consuming, highlighting the need for a fully automated, unsupervised machine learning method. Here, we aimed to develop an unsupervised clustering method with machine learning to automatically analyze fish behaviors by using fish 3D coordinates. To evaluate the performance of this method, five different datasets, including (1) interspecies fish that consisted of three fish species, which were *Danio rerio*, *Oryzias woworae*, and *Kryptopterus vitreolus*, (2) shoal sex composition, (3) caudal fin amputation effect, (4) habituation time effect, and (5) acute ethanol exposure effect on zebrafish behaviors, were analyzed. The current method utilizes multiple clustering algorithms, including K-Means, BIRCH, Gaussian Mixture Model, and Spectral Clustering, which were then applied to the 3D trajectory data in every dataset. Afterward, the clustering performance was evaluated using the Silhouette Score and Davies–Bouldin Index, followed by the use of deep-learning autoencoders to improve latent feature representation and enhance cluster separation. The clustering outcomes were further compared with predefined experimental groups using ARI and NMI and were qualitatively interpreted alongside conventional behavioral endpoints. External agreement varied among datasets and clustering algorithms. The proposed method can characterize differences in zebrafish 3D locomotor trajectory patterns across experimental conditions, while its separation power may decrease when behavioral differences are subtle or highly overlapping. Furthermore, the present study evaluated the method using multiple behavioral datasets; a similar framework can be extended to zebrafish models of neurodegenerative disease to identify disease-associated alterations in movement patterns and neurobehavioral organization. Finally, a user-friendly software package with easy installation and readable trajectory-analysis outputs was also developed that can be useful in biomedical research.

## Simple Summary

Zebrafish are widely used to study neurobehavioral changes because their locomotor patterns can reflect alterations in motor control, habituation, stress response, and responses to environmental stimuli. In this study, we developed an unsupervised machine-learning-based software package for analyzing three-dimensional (3D) fish trajectory data of three fish species, having major focus on zebrafish. The method was used to evaluate five datasets of fish behaviors, which included interspecific fish comparison, shoal sex composition, caudal fin amputation, habituation time, and acute ethanol exposure in zebrafish. Later, four clustering algorithms, including K-Means, BIRCH, Gaussian Mixture Model, and Spectral Clustering, were applied to analyze the 3D trajectory data. Afterwards, clustering performance was assessed using the Silhouette score and Davies–Bouldin index. In addition, to improve latent feature representation and enhance cluster separation, deep-learning autoencoders were then applied. Finally, the clustering outcomes were compared with the manually calculated behavioral endpoints, and the results showed consistency with conventional behavioral measurements. In summary, this study may give technical insights into neurodegenerative disease modeling with neural dysfunction affecting locomotion, motor coordination, habituation, and behavioral responses. Although neurodegenerative models were not applied directly for testing, the framework can be extended to identify disease-associated changes in zebrafish movement patterns. The key features of the study include a comprehensive setup developed with easy installation, readable outputs, and CPU/GPU compatibility to perform 3D trajectory big-data analysis.

## 1. Introduction

Animal behavior represents an observable output of neural, sensory, motor, and physiological processes. Therefore, quantitative behavioral analysis is widely used to study how animals respond to internal states and changing environmental conditions. While traditional image processing has laid the groundwork, recent advancements in video recording, animal tracking, and pose-estimation tools have made it possible to extract movement trajectories, posture, and other behavioral features from recorded videos [1]. These approaches allow researchers to extract trajectory-based features such as distance travelled, velocity, acceleration, turning angle, immobility, zone preference, depth position, and inter-individual distance. However, in recent years, artificial intelligence (AI) has played a significant role in medical image processing [2] and body scanning, as well as in computer engineering [3]. The recent hallmark of AI using machine learning (ML) approaches, such as supervised and unsupervised learning, has been demonstrated to increase the effectiveness of trajectory analysis by having critical advantages in terms of time and accuracy. Therefore, it is essential to develop non-supervised machine learning methods to obtain an accurate analysis of the animal behaviors, including fish.

Generally, fish behavior analysis is categorized into two-dimensional (2D) and three-dimensional (3D) trajectories that are retrieved from computer-vision-based programs [4]. 2D tracking is feasible and convenient enough to analyze fish behavior; however, certain limitations are observed in 2D trajectories compared to 3D, especially in motion behavior [5]. The interest in using 3D data for behavioral research is more conducive for researchers than 2D due to random behavioral shifts in animals, including rodents, insects, and primates [6,7]. Although several two-dimensional movement-analysis methods have been introduced for studying zebrafish behavior, 2D tracking [8] can only provide limited spatial information because zebrafish swim in a three-dimensional environment [9]. In particular, 2D views may underestimate or distort behavioral parameters such as swimming speed, peak speed, angular speed, wall-following, depth preference, vertical position, and spatial preference. Therefore, recent studies have increasingly used 3D tracking and trajectory-based analysis to obtain more accurate locomotor and behavioral measurements [10]. Furthermore, 3D tracking provides spatially realistic behavioral information and can reduce errors caused by projecting movement onto a two-dimensional plane [8]. It enables visualization and quantification of behavioral parameters such as 3D swimming distance, velocity, acceleration, angular velocity, turning angle, vertical position, depth preference, zone occupancy, path complexity, and time spent in different regions of the tank. These parameters are useful for evaluating locomotor activity, exploration, anxiety-like responses, social interaction, and stimulus-induced behavioral changes in zebrafish. Moreover, it has been evident that in the 2D locomotion trajectories, the behavior pattern is quite complex to understand with traditional methods while 3D trajectories provide a solid conclusion to refine the behavioral concerns in measuring fish locomotion in 3D (X, Y, and Z) trajectories. Therefore, a novel method in assessing 3D trajectories is always necessary to provide more reliable data regarding fish behaviors.

In recent years, various 3D locomotion analysis methods in adult zebrafish have been developed in recent years. For example, Etho Vision (Noldus, Inc., Leesburg, VA, USA) and ANYSys, Inc., (Blacksburg, VA, USA) [11] are commercially available software for tracking animal 3D locomotion, while open-source programs, Mouse Behavior Tracker [12], Animal Tracker [13], idTracker [14], and Janelia Automatic Animal Behavior Annotator (JAABA) [15,16] are also available. These programs work on different operating systems and have multiple features for tracking locomotion behaviors [17]. In addition, previous studies have demonstrated the usefulness of 3D locomotion analysis for zebrafish behavioural research. For example, Cachat et al. [8] applied 3D neurophenotyping to quantify zebrafish behavioural responses, while Stewart et al. [18] developed high-throughput 3D analysis for automated locomotor assessment. Audira et al. [4] further reported a single-camera approach for 3D locomotion analysis, although this method still required manual procedures. These studies show that 3D trajectory data can provide valuable behavioural information; however, some existing approaches still depend on manual or semi-automated analysis. Such procedures can be time-consuming and may introduce subjective variation. Therefore, automatic trajectory analysis supported by computational methods and artificial intelligence may improve consistency, reduce analysis time, and enhance the reliability of behavioural quantification. Crucially, even when 3D tracking is automated, the subsequent downstream analysis typically requires researchers to pre-define, extract, and calculate rigid behavioral endpoints (e.g., specific velocity thresholds or zone preferences). This reliance on pre-defined metrics means that subtle, complex, or novel behavioral patterns hidden within raw high-dimensional spatiotemporal trajectories are often overlooked. Unsupervised machine learning offers a powerful solution to this bottleneck by clustering raw trajectory data without human bias. However, raw 3D trajectory streams are highly complex and noisy, creating a clear need to investigate how advanced feature-extraction methods (like deep-learning autoencoders) can optimize raw data before clustering.

**Figure 1:**
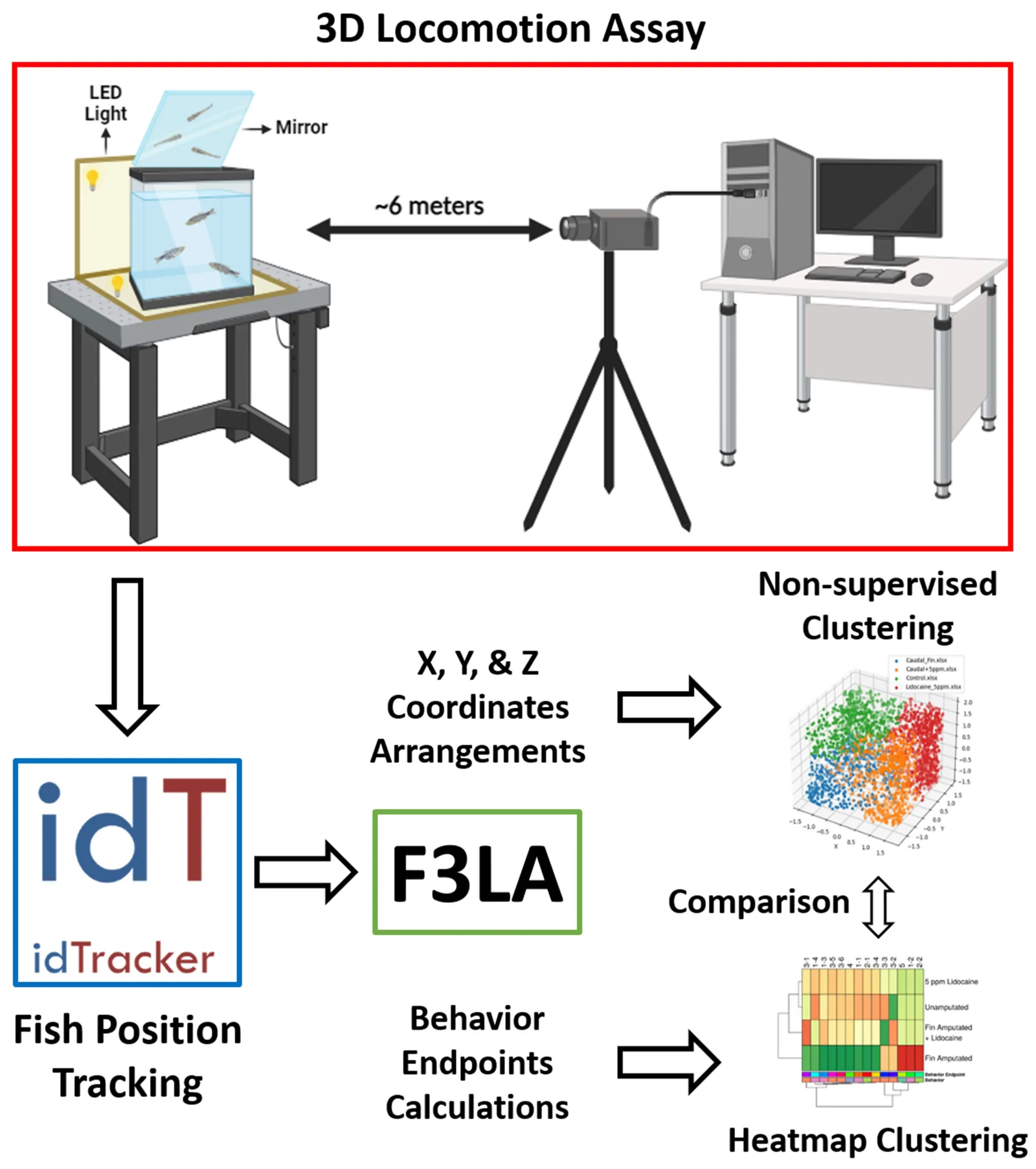
The overview of the current study. First, fish behaviors were recorded using the 3D locomotion assay setup (the image is taken from our prior publication [25]). Second, the recorded videos were analyzed by idTracker (version 2.1) to extract their coordinates. Subsequently, F3LA, our previously published APP to analyze fish behaviors, was used to arrange the generated coordinates and calculate several important behavior endpoints.

To address these limitations, the primary objective of this study was to develop and evaluate an automated pipeline capable of analyzing adult zebrafish 3D behaviors directly from raw spatial trajectories, thereby eliminating the requirement for manual endpoint calculation. The analysis was carried out on distinct 3D spatial trajectory patterns to see in the insights of movements of fish in closed tanks corresponding to the experimental conditions. Finally, this study aimed to verify if the automated clusters generated by these unsupervised methods align consistently with conventional, manually calculated biological endpoints. To achieve these goals, we developed a user-friendly, 3D trajectory-based clustering application that is able to generate output clusters using machine learning algorithms such as K-Means [19], balanced iterative reducing and clustering using hierarchies (BIRCH) [20], Gaussian mixture model (GMM) [21], and spectral clustering methods [22]. Later, the separation power of the generated clustering results, including intra-cluster and inter-cluster, was calculated using the Davies–Bouldin (DB) index [23] and Silhouette scores [24]. These metrics were used to quantify intra-cluster compactness and inter-cluster separation, rather than to directly establish biological validity. To improve the representation of trajectory patterns, deep-learning-based autoencoders were applied to generate a linear three-dimensional latent representation from the 3D trajectory data. The autoencoders were trained iteratively to minimize reconstruction error, and the resulting latent representations were then used to improve cluster separability across the four clustering methods. The generated clusters were further evaluated by comparing them with manually calculated behavioral endpoints from each dataset. These behavioral endpoints were summarized as heatmap data and used as reference information to assess whether the automated clustering patterns were consistent with conventional behavioral measurements. In addition, a graphical user interface (GUI)-based application was developed to facilitate user-friendly analysis of 3D trajectory data from different fish datasets and to provide quantitative outputs for locomotor and behavioral pattern analysis. The overview of the current study is shown in Figure 1.

## 2. Materials and Methods

### 2.1. Fish Maintenance

In the present study, three fish species were used: adult (6–8 months old) zebrafish (*Danio rerio*) with a body length and weight of ~3.5 cm and ~0.5 g, respectively; medaka (*Oryzias woworae*) with a body length and weight of ~3 cm and ~0.45 g, respectively; and glass catfish (*Kyrptopterus vitreolus*) with a body length and weight of ~7 cm and ~1.5 g, respectively. All of the fish were purchased from Zgenebio Inc. in Taipei, Taiwan (https://www.zgenebio.com.tw/ (accessed on 18 August 2024)), and reared in the circulated fish water system with a 14/10 h light/dark cycle for at least one month prior to the behavior tests. In the fish husbandry facility, the water with a pH of ~7.0, 300–1500 µS water conductivity, and a temperature of 26 ± 0.5 °C was filtered by UV light. The fish were fed with lab-grown brine shrimp and commercial dry food (Taiwan Hung Kuo Industrial Co., Ltd., Taipei, Taiwan), each once a day. The applied routine culture maintenance protocol was based on the method established by Avdesh et al. 2012 [26]. All animal experiments were conducted in accordance with the ARRIVE guidelines 2.0 [27]. The study protocol was approved by the Institutional Animal Care and Use Committee (IACUC) of Chung Yuan Christian University with Approval Number 112001.

### 2.2. 3D Locomotion Test and Fish Coordinates Extraction

In the current study, a 3D locomotion test was used to assess the differences in fish behavior patterns in different treatment groups [4]. Each behavioral recording analysis was performed on a shoal of six fish tested simultaneously. The independent behavioral recording (the shoal), rather than individual video frames or individual fish trajectories obtained from the same recording, was considered the biological replicate (*n*). Consecutive frames represent temporally repeated observations within a recording, and the six individual fish trajectories within the same recording were treated as subsampled observations belonging to the same experimental unit. Thus, experimental groups containing 18 fish comprised three independent recordings (6 fish × 3 recordings; *n* = 3), whereas groups containing 12 fish comprised two independent recordings (6 fish × 2 recordings; *n* = 2). For the novel-tank habituation experiment, three independent shoals were followed repeatedly across the specified observation intervals. Hence, the observations at different time points from the same shoal were treated as repeated measurements of the same biological replicates. Fish used in one experimental group were not reused in another experimental group.

This behavior test was conducted in an acrylic tank (20 × 20 × 20 cm) filled with ~6 L of filtered water, with a designed light-emitting diode (LED) light platform below and behind the tank to generate background light sources with high-contrast video and a mirror (30 × 30 cm) that was placed 45° from the top of the border tank to reflect the top view of the tank. Each test accommodated six fish in a single recording attempt by a Canon EOS 600D with a long-range zoom lens (EF-S 55–250 mm, Canon Inc., Tokyo, Japan) that was placed ~5 m in front of the test tank. The recorded video was at 50 frames per second with a resolution of 1280 × 720 pixels. In this study, we carried out five different experiments as described below.

#### 2.2.1. Interspecific Fish

In the first experiment, we assessed the behavioral performance differences between three fish species, which were zebrafish (*D. rerio*), medaka (*O. woworae*), and glass catfish (*K. vitreolus*), in the open field test. These three fish species were chosen considering that zebrafish and medaka are the two most used teleost models, especially in the behavioral research field, while the glass catfish was used due to its relatively bigger body size, to inspect the capability of the current method in evaluating fish behavior with different body sizes. For this experiment, a total of 18 fish from each group were used, with a total of 54 fish in a mixed-sex group. It is important to emphasize that the primary objective of this specific dataset was to evaluate the computational separation power of the clustering software using diverse input patterns, rather than to conduct a fundamental ecological comparison. While identical tank conditions ensured methodological consistency for the software, the fixed tank dimensions do not represent an ecologically equivalent environment for all three species due to differences in their body size, swimming mode, and preferred water layers. Therefore, the observed clustering patterns should be interpreted strictly as the algorithm’s ability to differentiate three-dimensional locomotor trajectories within a standardized assay space, rather than as direct evidence of ecological or behavioral similarity among the species.

#### 2.2.2. Shoal Sex Composition Effect on Zebrafish Behavior

Second, the effect of shoal sex composition on zebrafish locomotor behavior was evaluated in the 3D locomotion test by evaluating three fish groups: male only, female only, and male and female (1:1 ratio) [28]. Similar to the first experiment, a total of 18 fish per group were used (totaling 54 fish: 27 males and 27 females). Each recording contained six fish tested simultaneously. Because individuals were observed within a social group, the experiment was explicitly designed to assess the effect of shoal sex composition, which naturally encompasses group dynamics such as courtship and social structure, rather than to isolate intrinsic individual sex effects.

#### 2.2.3. Zebrafish Habituation in a Novel Environment

In experiment three, the habituation process of a group of zebrafish during the novel tank exposure was also evaluated. For this experiment, the fish behaviors were recorded for 1 min observation periods at 0–1, 5–6, 10–11, 15–16, and 20–21 in the novel test tank. A total of 18 fish in a mixed-sex group were used in this experiment.

#### 2.2.4. Acute Effects of Ethanol on Zebrafish Behaviors

For the fourth experiment, we also aimed to evaluate the performance of the current clustering method to assess the behavior differences in zebrafish after being acutely treated with low doses of ethanol, since it has been known to alter zebrafish behaviors in a dose-dependent manner [29]. The control group (18 fish, mixed sex) comprised three independent shoals (*n* = 3). The 0.25% and 1.0% ethanol-treated groups (12 fish each, mixed sex) comprised two independent shoals per treatment (*n* = 3). This reduction in sample size for the treatment groups was deliberately applied to adhere to the 3Rs principle (Reduction), because the locomotor effects of acute ethanol in zebrafish are well-documented; fewer replicates were required to confirm the phenotype [27]. We acknowledge that this unbalanced experimental design yields an unequal number of total spatial observations between groups, which may influence cluster-size-sensitive internal validation indices. Therefore, the trajectory-based clustering outcomes for this dataset are interpreted alongside conventional behavioral endpoints.

#### 2.2.5. Caudal Fin Amputation Effects on Zebrafish Behaviors

The fifth experiment consisted of unamputated, fin-amputated, fin-amputated +5 ppm lidocaine, and 5 ppm lidocaine only groups. However, for the current manuscript, only 1 h post-amputation data were used since, based on our prior study, this time interval showed more robust behavior pattern differences between groups, as indicated by the lower locomotion and movement complexity, as well as the altered exploratory behaviors [30]. Here, the fish were divided into four groups: unamputated, caudal fin-amputated, caudal fin-amputated with 5 ppm of lidocaine, and 5 ppm of lidocaine groups, with each group consisting of 18 fish, with 6 fish in each replication.

For all of the experiments, except the time chronology experiment (novel tank test), the fish behaviors were recorded for 5 min. Afterward, all of the recorded videos were analyzed using idTracker (http://www.idtracker.es/, accessed on 9 August 2023) to extract each fish’s X and Y coordinates from the top view and Y and Z coordinates from the side view in every frame [14]. Up to this point, the applied protocol was based on the prior publication [4]. Finally, F3LA, a Python-based tool, was used to accurately compare the aligned sequence and calculate the X, Y, and Z coordinates of each fish, which were later used for the clustering process [31]. Furthermore, this tool was also used to extract several important behavior endpoint parameters in each experiment to help in evaluating the clustering results by comparing them with the behavior endpoint results as the standard reference that had been used to evaluate fish behaviors. However, one has to keep in mind that since the unsupervised clustering results and conventional behavioral endpoints were derived from the same underlying trajectory recordings, the agreement between these analyses serves to compare analytical methods, rather than serving as independent biological validation. Subsequently, these behavior endpoint data were also clustered by using the hierarchical clustering method by ClustVis (https://biit.cs.ut.ee/clustvis/ (accessed on 19 June 2026)) to reduce the data dimension and complexity. The detailed information regarding the calculated behavior endpoints is described in Table S1. All of the statistical analyses and graphs were conducted and drawn, subsequently, by either GraphPad Prism (GraphPad Software version 8 Inc., La Jolla, CA, USA) or OriginPro v.9.3 (OriginLab Corporation, Northampton, MA, USA).

### 2.3. Data Preprocessing

Before delving into clustering analysis, a rigorous data preprocessing phase was executed to ensure the quality and integrity of the self-generated fish dataset. The dataset underwent several crucial steps to enhance its suitability for subsequent analysis. First, the dataset was examined for outliers, which can distort clustering results, using Z-Score-based outlier removal using Z-Score [32]. The Z-Score method was employed to identify and eliminate outliers that fell outside a defined threshold. This process safeguarded the dataset from skewed patterns due to extreme values. Next, normalization with a standard scaler was also used to ensure equitable data treatment across different scales. This normalization procedure transformed the dataset’s features to a standardized range, reducing the influence of differing magnitudes on clustering outcomes. All of the pre-processing steps were carried out on a high-power computer system to execute processing faster.

### 2.4. Python Libraries Used for Clustering

The current study utilized the Python programming language [33] to construct the 3D clusters for each zebrafish’s X, Y, and Z coordinates in each dataset. Initially, the data were saved in a Microsoft Excel format [33] and underwent data pre-processing that was performed using a previously published method by Usman et al. [34], in which the scripts were written to perform data pre-processing and to generate 3D clusters on a given dataset. The Python-based libraries such as pandas (https://pandas.pydata.org/, accessed on 22 June 2023), matplotlib (https://matplotlib.org/, accessed on 22 June 2023), scikit-learn (https://scikit-learn.org/stable/, accessed on 22 June 2023), and openpyxl (https://openpyxl.readthedocs.io/en/stable/, accessed on 23 June 2023) were used to read and manipulate the 3D zebrafish trajectories data from Excel files and model the clusters using K-Means, BIRCH, Mini Batch K-Means and Gaussian mixtures algorithms. Essential libraries were installed using the Python Package Installer (pip) (https://pypi.org/project/pip/, accessed on 22 June 2023). All computations were performed in a Jupyter notebook environment with an Intel^®^ Core™ i9-9900KF CPU @ 3.60GHz and a 32 GB RAM system [35]. Due to the large dataset of trajectories, we used an NVIDIA GeForce GTX 1060 with 6 GB of memory as a graphical processing unit (GPU) to perform random clustering that generated multiple clusters to show differences in each data group. The coding loops were generated for each input file, and the fish data per group were merged into a single Excel sheet within the respective data frame. Afterward, the data were normalized to ensure consistency, and the combined DataFrame was then saved in the output folder to show the original data labeling. This process was repeated for each trajectory data group to perform further clustering functions.

### 2.5. Clustering

The original clustering analysis used single 3D coordinate observations X, Y, Z corresponding to the spatial position of an individually tracked fish in a given video frame. Six fish were recorded simultaneously during each recording session; however, the six-fish shoal was not treated as a single collective trajectory. In the original analysis, frame-level XYZ observations from the relevant experimental groups were processed independently of temporal order. Therefore, these clusters primarily represent recurrent spatial occupancy and distribution patterns within the test environment rather than temporally ordered locomotor behavior. To complement this spatial-occupancy analysis, a separate trajectory-level temporal analysis was performed. Continuous trajectory windows were generated within each recording while preserving the original frame order. The primary analysis used 100-frame windows with 50% overlap between consecutive windows. Each window contained the X, Y, and Z coordinates together with derived movement features, including speed, acceleration, turning angle, and vertical displacement (ΔZ). Three feature representations were evaluated as raw trajectory-window features, PCA-transformed features, and a Conv1D autoencoder latent representation. Each representation was evaluated with K-Means, BIRCH, Gaussian Mixture Model, and Spectral Clustering. Robustness to window duration was further evaluated using 25, 50, 100, and 200-frame windows, with the alternative window lengths treated as sensitivity analyses. Training/validation partitioning was performed at the whole-recording stage so that overlapping windows from the same recording should not occur in both partitions.

Both the spatial-occupancy and trajectory-level analyses were performed using unsupervised machine-learning clustering methods (https://scikit-learn.org/stable/modules/generated/sklearn.cluster.KMeans.html, accessed on 30 June 2023). The resulting cluster assignments were saved for subsequent evaluation and visualization. 3D scatter plots were generated using matplotlib and mpl_toolkits. mplot3d (https://matplotlib.org/mpl_toolkits/, accessed on 1 July 2023) to visualize the clustering results. In addition, a graphical user interface was developed using the Python tkinter library version 8.5 to facilitate application of the clustering workflow to user-provided datasets. A detailed tutorial regarding the installation and usage of the APP is provided at the GitHub link https://github.com/Alizebrafish/3D_Clustering (accessed on 23 September 2026).

#### 2.5.1. K-Means Clustering

This clustering approach determines and specifies the initial number of clusters *k*. The centers of these clusters were chosen randomly and then moved toward an optimal point. The data points are allocated to each cluster based on the Euclidean distance between them using the following relationship [35]:

$$\left(x_{i},\;c_{i}\right)=\;{\left\|x_{i}-c_{j}\right\|}^{2}$$

where $x_{i}$ is the $i^{th}$ data point contained in the data, c is the center of $j^{th}$ cluster, and $\left\|.\right\|$ represents the Euclidean distance between x and c. After each iteration, the centers of clusters are recalculated to minimize the sum of all Euclidean distances between data points and centers, represented as follows [36]:

$$c_{j}=\frac{1}{n_{j}}\sum_{i=1}^{n}x_{i}$$

The weighted sum between the clusters is minimized to determine the convergence point. The within-cluster sum of squares (*WCSS*). Mathematically, it is represented as follows [36]:

$$WCSS\left(k\right)=\;\sum_{i=1}^{n}\sum_{j=1}^{k}{\delta \left(i,j\right)\left\|x_{i}-c_{j}\right\|}^{2}$$

$\delta \left(i,j\right)$ is the indicator function whose value can be 1 or 0 depending on the data.

#### 2.5.2. BIRCH Clustering

Balanced Iterative Reducing and Clustering using Hierarchies (BIRCH) algorithm is another clustering approach that uses branching factors and thresholds for splitting and allocating the data clusters. A CF tree is created using a hierarchical structure that determines the distribution of the data points and their corresponding overlapping clusters. The clusters are then selected and re-created to reach an optimal point. In the CF node creation, the number of data points N is chosen along with linear and squared sums of data points represented as LS  and SS, respectively. The tree nodes T are updated upon each insertion of data points using the following equations [37]:

$$N_{new}=N_{old}+1$$

$$LS_{new}=LS_{old}+x$$

$$SS_{new}=SS_{old}+x.x$$

The model then passes through splitting and global clustering. Finally, the quantification function is applied as follows:

$$Q=\frac{N.SS-LS.LS}{N.(N-1)}$$

#### 2.5.3. Gaussian Mixture Model (GMM)

In this clustering method, the number of clusters k is chosen to fit the data. The mean, covariance, and mixing coefficients are computed for the Gaussian components, followed by an Expectation-Maximization algorithm that updates and optimizes the model parameters. The EM objective function is represented as follows [38]:

$$\log p\left(X|\;\theta \right)=\;\sum_{i=1}^{N}\log\left(\sum_{j=1}^{k}\pi_{j}\;.\;\;f\left(x_{i}|\;\mu_{j},\;\sum j\right)\right)$$

Here N is the number of data points, θ is the model parameter, $\pi_{j}$ is the $j^{th}$ mixing component, and $f\left(x_{i}|\;\mu_{j},\;\sum j\right)$ is the probability density function of the $j^{th}$ component.

#### 2.5.4. Spectral Clustering

This technique transforms data into a lower dimension using eigenvalues and Eigenvectors. We generated an Affinity matrix A that calculates and appends the similarities between the data points pairwise. Various methods, including k-nearest neighbor [36] or Gaussian similarity [37], can be used; however, the Radial Basis Function (RBF) was used in this case. Finally, the method uses K-Means on the transformed data to create clusters. The sequence of steps involves the generation of an affinity matrix A followed by the computation of the degree matrix D where the diagonal $D_{ii}$ represents the sum of affinities. The degree matrix follows the Laplacian matrix [39].

L=D−A. L

was then normalized as follows:

$$L_{norm}=D^{-1/2}LD^{-1/2}$$

Finally, eigen composition takes place by finding the eigenvalues and eigenvectors, and after selecting a specific number of eigenvalues, the K-Means clustering was applied as explained above.

#### 2.5.5. Deep Learning Auto-Encoder Implementation

**Figure 2:**
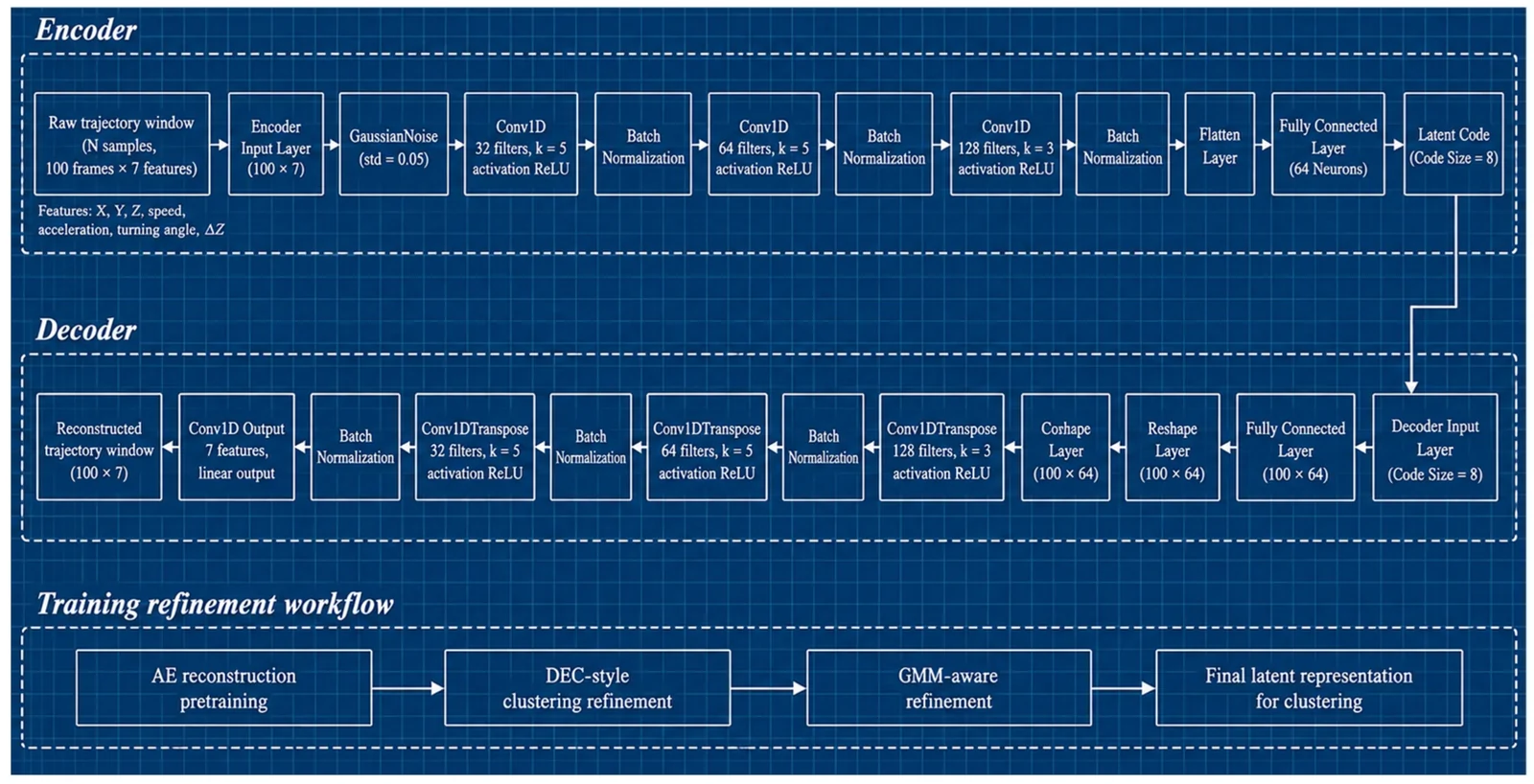
The architecture of the deep learning autoencoder applied in the study.

The clustering performance on the original standardized XYZ representation showed limited separation in several datasets; a convolutional autoencoder was applied to learn a nonlinear three-dimensional latent representation prior to clustering. Autoencoders play a crucial role in clustering applications, showcasing their effectiveness in unsupervised learning tasks. The architecture of autoencoders is presented in Figure 2. The Conv1D autoencoder used encoder convolutional layers with 32, 64, and 128 filters and kernel sizes of 5, 5, and 3, respectively, with same padding. The encoder output was flattened, passed through a 64-unit dense layer, and compressed into an 8-dimensional latent representation. The decoder used transposed convolutional layers with 128, 64, and 32 filters. The model was trained using the Adam optimizer with an initial learning rate of 1 × 10^−4^, mean squared error loss, a maximum of 150 epochs, and a batch size of 64. Early stopping was applied with a patience of 15 epochs and restoration of the best model weights, while the learning rate was reduced by a factor of 0.5 after 6 epochs without improvement. A fixed random seed of 55 was used to improve reproducibility. The graphical presentation of the autoencoder architecture in Figure 2 was prepared with the assistance of Google Flow using the Nano Banana [40] image generation model and was manually verified against the implemented model architecture.

The fundamental process of the autoencoder involves converting and subsequently interpreting input data and reconstructing the original input while acquiring a meaningful representation [41]. When discussing clustering with autoencoders, we can represent the input data as *X*, the encoder function as *E* (⋅), the decoder function as *D* (⋅), and the reconstructed data as *X*′. The autoencoder encodes the learned latent representation as *Z*. The fundamental equations for autoencoders can be expressed mathematically as:

Encoding: Z=E(X)

$$\mathrm{Decoding}:\;X^{\prime }=D(Z)$$

The goal during training is to decrease the reconstruction error, which is commonly assessed using a loss function *L*. This is accomplished by improving the parameters of both the encoder and decoder. The loss function is determined by measuring the discrepancy between the original data *X* and the rebuilt data *X*′:

$$Loss\;Function=L(X,X^{\prime })$$

The latent representation *Z* acquired from the encoder can be utilized for clustering purposes in clustering applications. The developed representation retains inherent patterns and structures in the data, enabling efficient clustering based on similarity in the latent space [42].

For autoencoder input, each trajectory sample was represented as a continuous window of 100 consecutive frames with seven standardized features per frame: X, Y, Z coordinates, speed, acceleration, turning angle, and vertical displacement (ΔZ). Thus, the autoencoder input tensor had the shape *n* × 100 × 7, where *n* denotes the number of trajectory windows. In this representation, the dimension of length 100 corresponds to consecutive temporal frames, while the seven feature channels describe spatial position and derived locomotor characteristics. The Conv1D layers were therefore applied along the temporal dimension to extract local movement patterns from the ordered trajectory sequence.

### 2.6. Separation Power Evaluation

Finally, the DB index score was calculated to evaluate the quality of the clusters constructed by selected models [23]. This index indicates the average similarity between each cluster and its most similar cluster, with lower scores indicating better separation and more distinct clusters. The index is computed as follows for each cluster [43]:

DB_i = (1/C) ×∑(R_i + R_j)/M_i,j

where C is the total number of clusters, R_i is the average distance from the centroid of cluster i to all points in the cluster, and M_i, j are the distances between the centroids of clusters i and j.

In addition, the overall performance of the four selected models was evaluated by calculating Silhouette scores [24]. When applied to measure the overall comparison of four methods, this score shows how similar each data point is in different clustering patterns. Silhouette score values vary from −1 to 1, with 1 where a higher score indicates better-defined clusters; 0.5 is considered the threshold range when evaluating clustering data [24,44]. Silhouette scores were computed as:

s_i = (b_i − a_i)/max(a_i, b_i)

where i is the average distance from point i to other points in the same cluster; i is the smallest average distance from point i to points in a different cluster.

To ensure that all features contribute equally to the clustering process, the standardization process was conducted, and the Computed Covariance Matrix was utilized to calculate the covariance matrix of the standardized data. Subsequently, by computing Eigenvectors and Eigenvalues, we found the eigenvectors and eigenvalues of the covariance matrix. Afterward, the eigenvalues were sorted in descending order, and the top K eigenvectors were chosen. Later, the original data were projected onto the selected eigenvectors to obtain a lower-dimensional representation, as the covariance matrix Σ was computed as [45]:

Σ = (1/N) × ∑((x_i − μ) × (x_i − μ)^T)

where N is the number of data points, and μ is the mean vector. The Eigenvectors and Eigenvalues, v and λ, are obtained by solving the eigenvalue problem:

Σ × v = λ × v

where v is the eigenvector and λ is the corresponding eigenvalue. Finally, the data were projected into the selected eigenvectors to obtain the lower-dimensional representation [46]:

x′ = x × V

where x′ is the projected data, x is the original data, and V is the matrix of selected eigenvectors.

### 2.7. Data Incorporation

In the initial stage, a self-generated zebrafish dataset was subjected to an exploratory analysis through multiple clustering methods to uncover inherent patterns and groupings within the data. As mentioned above, four distinct clustering algorithms (K-Means, BIRCH, Gaussian Mixture Models (GMM), and Spectral Clustering) were selected. K-Means clustering aims to partition data points into K clusters, where each data point belongs to the cluster with the nearest mean. The algorithm involves the initialization of randomly selected K initial cluster centroids. Afterward, each data point was assigned to the closest centroid, and new centroids based on each cluster’s mean of data points were calculated. These steps were repeated until convergence was achieved. For each data point x_i, the nearest centroid c_j was found using Euclidean distance [47]:

j = argmin ||x_i − c_j||^2

followed by a centroid update (c_j_) by calculating the mean of all data points assigned to cluster j:

c_j = (1/N_j) × ∑(x_i) for all x_i in cluster j

The initialization was performed by loading and preprocessing the dataset, which contains 3D labels with their respective tracking groups, that were collected from multiple Excel files and combined into a single dataset. Further, each method builds a clustering feature (CF) tree by recursively clustering data points that assist in summarizing the data by forming a hierarchical structure. Each non-leaf node of the tree represents a cluster and contains N data points in the sub-cluster, LS the linear sum of data points, and SS the squared sum of data points. Finally, the CF tree nodes were merged to form clusters at different levels.

### 2.8. Distance Between Nodes Determination

Next, the distance between two CF nodes was calculated using the following formula [48]:

Distance (CF1, CF2) = sqrt ((LS1 − LS2)^2 + (LS1 − LS2)^2 + (LS1 − LS2)^2)

This calculation was essential to merge the closest nodes in order to identify the pair of CF nodes with the minimum distance and subsequently merge them into a new CF node. Afterward, the new CF node’s N, LS, and SS values were updated, followed by removing the connected CF nodes from the CF tree and adding the new CF node. The merging process was repeated until the desired number of clusters was achieved. The CF tree results provided a hierarchical representation of clusters, allowing for efficient and balanced clustering.

Next, GMM clustering assumes that data points are generated from a mixture of several Gaussian distributions. The algorithm involves K Gaussian components with random means, covariances, and weights, with an Expectation-Maximization (EM) algorithm that computes the posterior probabilities of data points belonging to each element. Here, the means, covariances, and consequences were updated based on the expected responsibilities, and the probability density of the Gaussian distribution was expressed as follows [49]:

P(x | μ, Σ) = (1/(2π)^{d/2} × |Σ|^{1/2}) × exp(−0.5 × (x − μ)^T × Σ^{−1} × (x − μ))

Similarly, spectral clustering aims to partition data points based on the eigenvectors of the Laplacian matrix of a similarity graph. The algorithm constructs an affinity matrix based on pairwise similarities between data points. It is often constructed using a Gaussian kernel to measure the similarity between data points. Given a dataset with N data points x_1_, x_2_, …, x_N, the affinity matrix W is computed as:

W[i, j] = exp(−||x_i − x_j||^2/(2 × σ^2))

where

W[i, j] is the affinity matrix entry at row i and column j.

||x_i − x_j|| is the Euclidean distance between data points x_i and x_j.

σ is a parameter that controls the width of the Gaussian kernel.

The Laplacian matrix L is derived from the affinity matrix W. There are different ways to define the Laplacian matrix. However, one common choice is the unnormalized Laplacian, which is computed as:

L = D − W

where D is a diagonal matrix (degree matrix), D[i, i] is the sum of the weights in the i-th row (i.e., the sum of similarities for data point x_i), and W is the affinity matrix.

### 2.9. Eigenvectors of the Laplacian Computation and Their Usage in the Clustering Process

Next, the eigenvalues and eigenvectors of the Laplacian were computed to find the first K smallest eigenvectors that correspond to the smallest eigenvalues. These eigenvectors provided a lower-dimensional data representation that captures the underlying structure. Once the eigenvectors were calculated, traditional clustering algorithms (such as K-Means) were used to group similar points on the transformed data.

Affinity Matrix: The entry W[i, j] is computed using the Gaussian kernel:

W[i, j] = exp(−||x_i − x_j||^2/(2 × σ^2))

The Laplacian matrix L was obtained by subtracting the affinity matrix from the degree matrix:

L = D − W

The eigenvalues and eigenvectors were computed by solving the eigenvalue problem for the Laplacian matrix L:

L × v = λ × v

where v is the eigenvector and λ is the corresponding eigenvalue. By performing spectral clustering on the transformed data using the eigenvectors, the algorithm partitioned the data into clusters based on the underlying structure revealed by the lower-dimensional representation.

### 2.10. Number of Clusters

For each experimental dataset, trajectory windows from all experimental groups were pooled before clustering. Clustering was therefore not performed separately within individual experimental groups. The number of clusters (k) was predefined according to the number of experimental conditions represented in each dataset. Accordingly, k = 3 was used for the interspecies dataset, k = 3 for the shoal sex-composition dataset, k = 4 for the caudal-fin-amputation dataset, k = 5 for the habituation dataset, and k = 3 for the acute ethanol dataset. The same predefined value of k was applied to both the raw trajectory representation and the autoencoder-derived latent representation within each dataset. This ensured that comparisons of the Silhouette score, DB index, and cluster separation before and after autoencoder transformation were performed under an identical cluster-number configuration. K-Means, BIRCH, Gaussian Mixture Model, and Spectral Clustering were applied separately to each dataset representation. Experimental group labels were not used as clustering input features and were retained only for post hoc interpretation and the calculation of Adjusted Rand Index (ARI) and Normalized Mutual Information (NMI) [50]. Therefore, the Silhouette score and Davies–Bouldin index describe the internal geometry of the complete pooled clustering solution for each dataset and do not represent separate clustering analyses performed within individual experimental groups. Additionally, elbow metrics were generated as descriptive visualizations of possible cluster structures for both the raw trajectory representation and the autoencoder-derived latent representation. The number of elbow points was evaluated by minimizing the within-cluster sum of squares (*WCSS*). Mathematically, it is represented as follows:

$$WCSS\left(k\right)=\;\sum_{i=1}^{n}\sum_{j=1}^{k}{\left\|x_{i}-c_{j}\right\|}^{2}$$

Here, n represents the number of data points, k represents the number of clusters (ranging between 1 and 11 in the present case), $x_{i}$ is the $i^{th}$ data point contained in the data, c is the center of $j^{th}$ cluster, and $\left\|x_{i}-c_{j}\right\|$ represents the Euclidean distance between $x_{i}$ and $c_{j}$. These plots were not used to determine the final number of clusters k, which was predefined according to the number of experimental groups in each dataset as detailed above.

To enhance the clustering results, a sophisticated deep-learning approach in the form of an auto-encoder model was introduced. This model combined a Convolutional Neural Network (CNN) [51] and dense layers to capture intricate features and relationships within the data. The architecture of the auto-encoder [41] involves two 1D CNN layers with 16 and 32 filters, followed by batch normalization and flattening, with a dense layer to extract essential features and dense layers to transform the data into the desired shape. The encoded representation obtained from this process is then passed through thick layers, effectively forming the encoder portion of the auto-encoder. Mathematically, the encoder part can be represented using the following taxonomy.

$$Z_{1}=f(X\;*\;W_{1}+b_{1})$$

$$Z_{2}=f\left(Z_{1}\;*\;W_{2}+b_{2}\right)$$

$$Z_{3}=GlobalMaxPooling(Z_{2})$$

$$Z_{4}=f\left(Z_{3}.W_{d}+b_{d}\right)$$

Here, X represents the input data, while $W_{1}$ and $W_{2}$ are the weights of the first and second convolutional layers, respectively. Meanwhile, b represents the bias for each layer, and *f* is the relu activation function, whilst ∗ and . represent convolution and matrix multiplication, respectively. The decoder uses the following mathematical equations, which are presented as dc:

$$Z_{5}=f\left(Z_{c}.W_{dc}+b_{dc}\right)$$

$$Z_{6}=f\left(Z_{5}.W_{6}+b_{6}\right)$$

$$Z_{7}=f\left(Z_{6}.W_{7}+b_{7}\right)$$

The number of clusters k was predefined according to the number of experimental conditions represented in each dataset. Accordingly, k = 3 was used for the interspecies dataset, k = 3 for the shoal sex-composition dataset, k = 4 for the caudal-fin-amputation dataset, k = 5 for the habituation dataset, and k = 3 for the acute ethanol dataset. The same value of k was applied to both the raw trajectory representation data and the autoencoder-derived latent representation within each dataset. This ensured that comparisons of Silhouette score, DB index, and cluster separation before and after autoencoder transformation were performed under an identical cluster-number configuration. Experimental group labels were not used as clustering features and were retained only for interpretation of the results based on clustering. Additionally, ARI and NMI computations were performed to validate the method. Elbow metrics were generated as descriptive visualizations of possible cluster structure for both the raw trajectory representation and the autoencoder-derived latent representation. These plots were not used to determine the number of clusters k, which was predefined according to the number of experimental groups in each dataset. The corresponding trajectory-level elbow plots before and after autoencoder transformation.

## 3. Results

**Figure 3:**
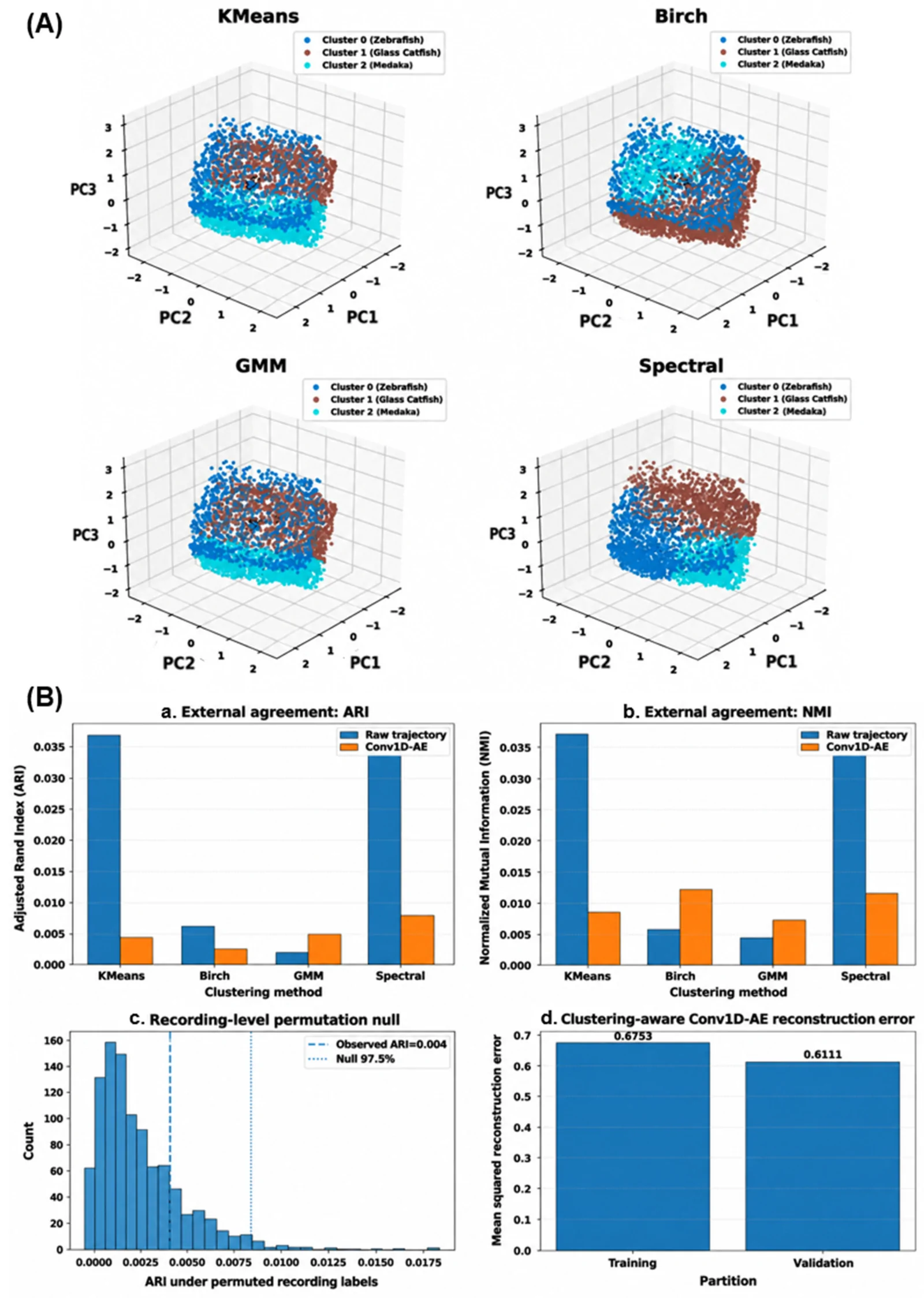
(**A**) Clustering results for the interspecies trajectory dataset after autoencoder transformation using K-Means, BIRCH, Gaussian Mixture Model (GMM), and Spectral Clustering with k = 3. Colors indicate the unsupervised cluster assignments. Experimental species labels were not used to generate the clusters and were used only for mapping and external agreement analysis. (**B**) Quantitative evaluation of clustering performance. (**a**) ARI and (**b**) NMI compare the unsupervised cluster assignments with the predefined species groups for the raw trajectory and Conv1D-autoencoder representations. (**c**) The recording-level permutation analysis shows the null distribution of ARI obtained by shuffling experimental labels at the recording level. (**d**) The reconstruction panel shows the mean squared reconstruction error for the training and held-out validation recordings.

**Figure 4:**
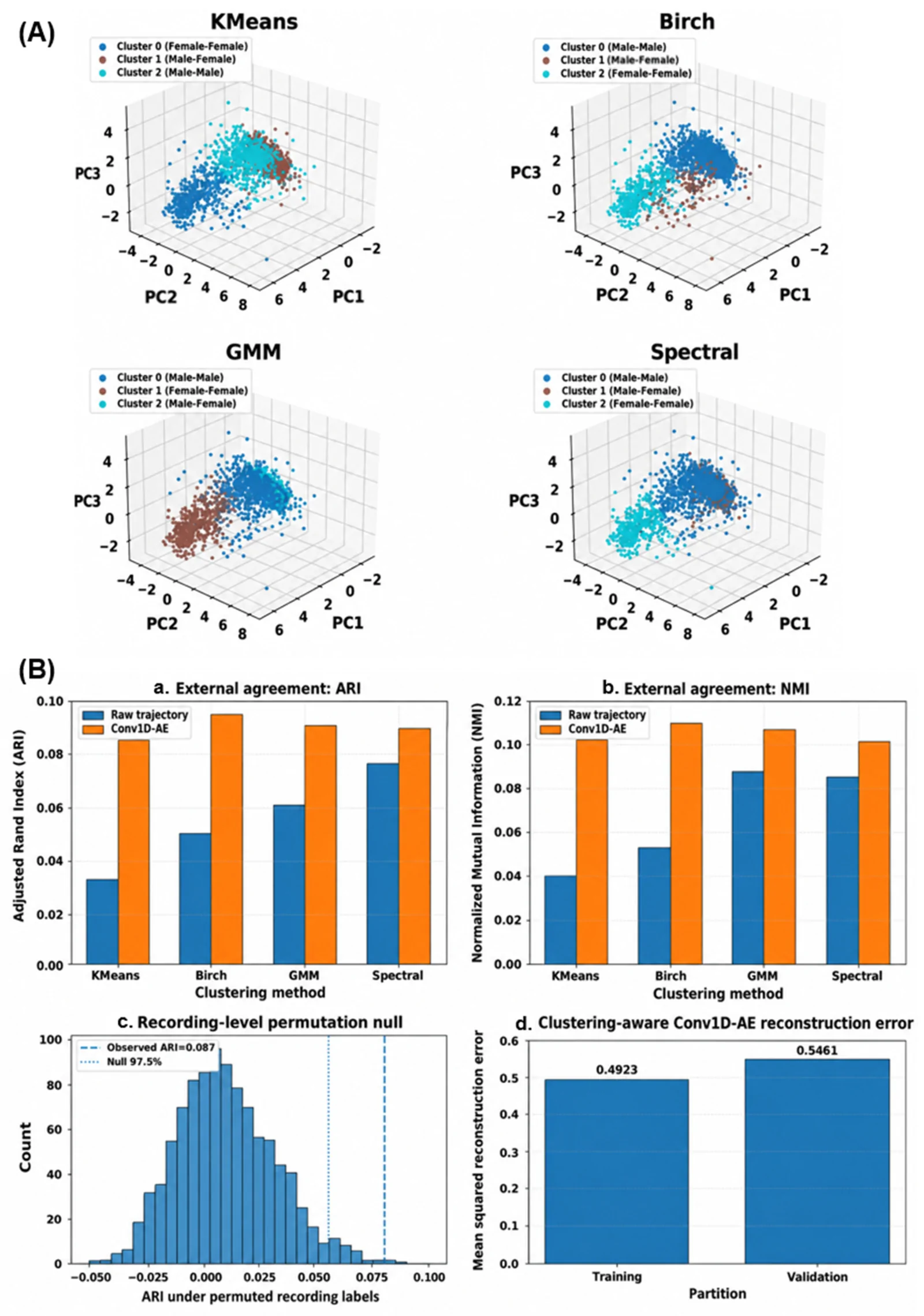
(**A**) Clustering results for the shoal sex-composition trajectory dataset after autoencoder transformation using k = 3. Colors indicate unsupervised cluster assignments. Experimental group labels were not used to generate the clusters and were used only for mapping the interpretation and external agreement analysis. (**B**) (**a**) ARI and (**b**) NMI comparison for all-male, all-female, and mixed-sex shoals. (**c**) The recording-level permutation analysis shows the null distribution of ARI obtained by shuffling experimental labels at the recording level. (**d**) The reconstruction panel shows the mean squared reconstruction error for the training and held-out validation recordings.

**Figure 5:**
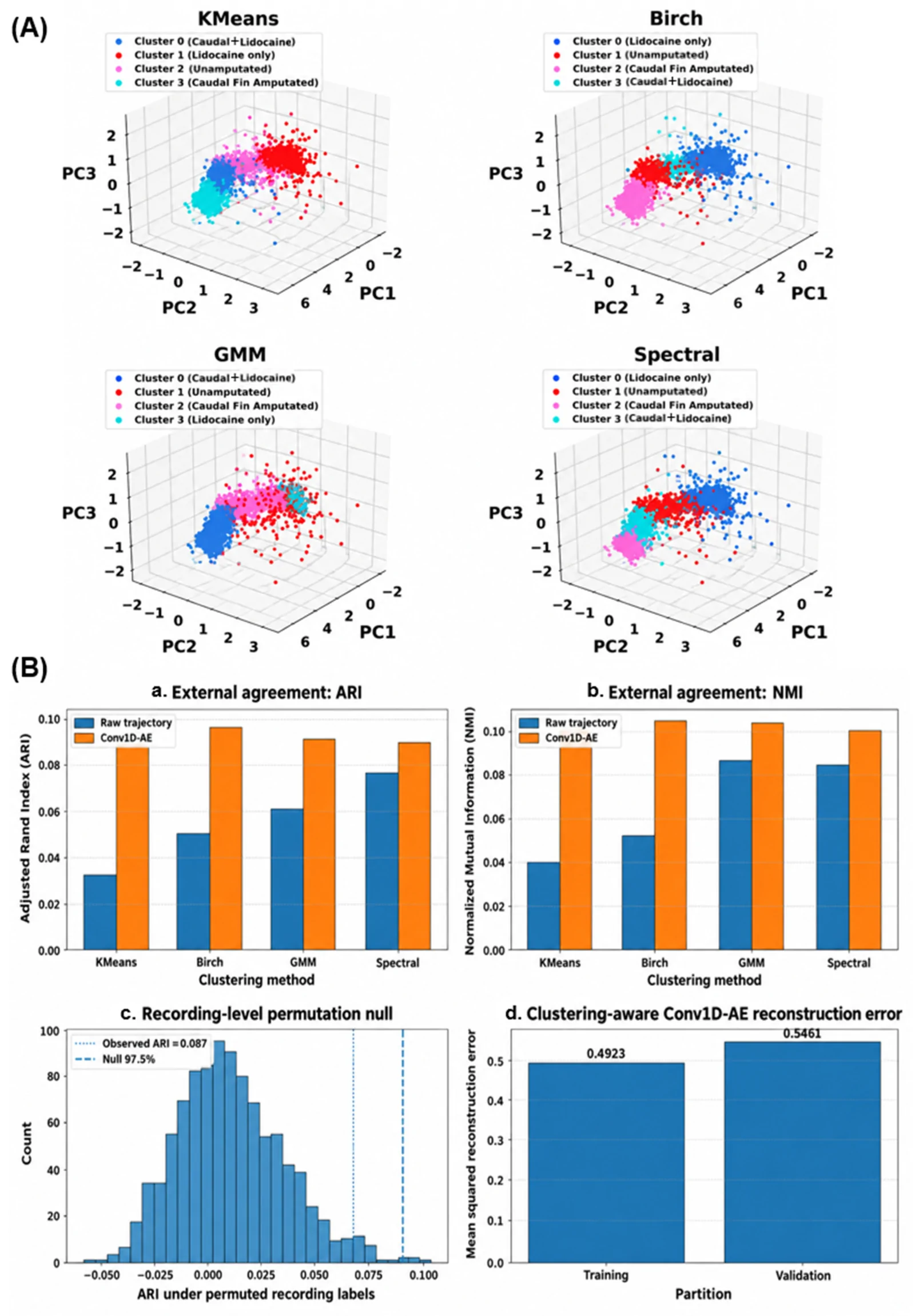
(**A**) Clustering outcomes for the caudal-fin-amputation dataset after autoencoder implementation using k = 4. Colors indicate unsupervised clusters rather than predefined treatment labels. (**B**) (**a**) ARI and (**b**) NMI comparison for the fin amputation dataset. (**c**) The recording-level permutation analysis shows the null distribution of ARI obtained by shuffling experimental labels at the recording level. (**d**) The reconstruction panel shows the mean squared reconstruction error for the training and held-out validation recordings.

**Figure 6:**
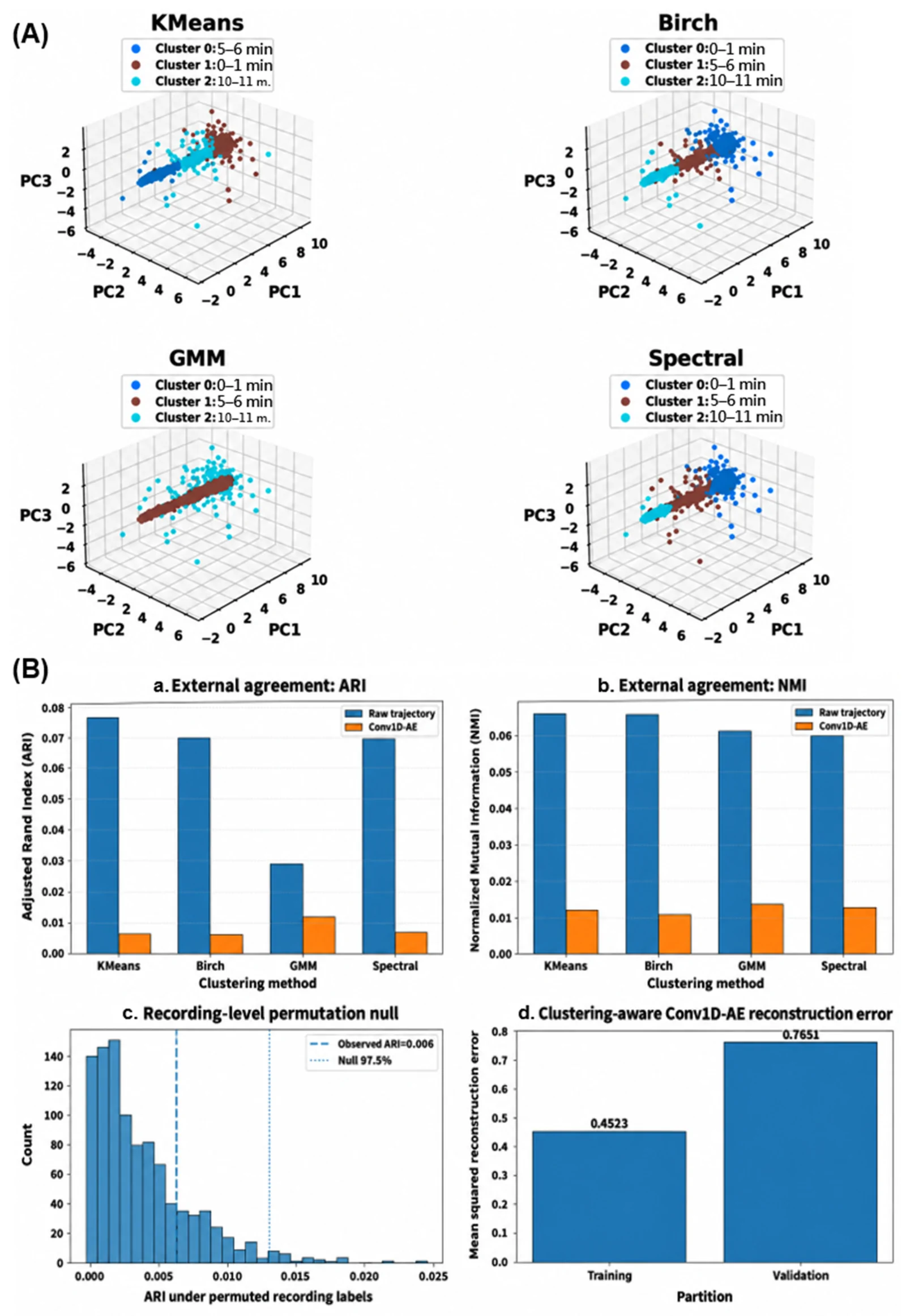
(**A**) The habituation dataset-based clustering analysis after autoencoder, using k = 5. The five observation intervals were retained as experimental labels only for respective interpretation. (**B**) (**a**) ARI and (**b**) NMI comparison for 3 intervals of data during one 1 min observation period, with 5 min time intervals between each observation period until 21 min after exposure to the novel tank. (**c**) The recording-level permutation analysis shows the null distribution of ARI obtained by shuffling experimental labels at the recording level. (**d**) The reconstruction panel shows the mean squared reconstruction error for the training and held-out validation recordings.

**Figure 7:**
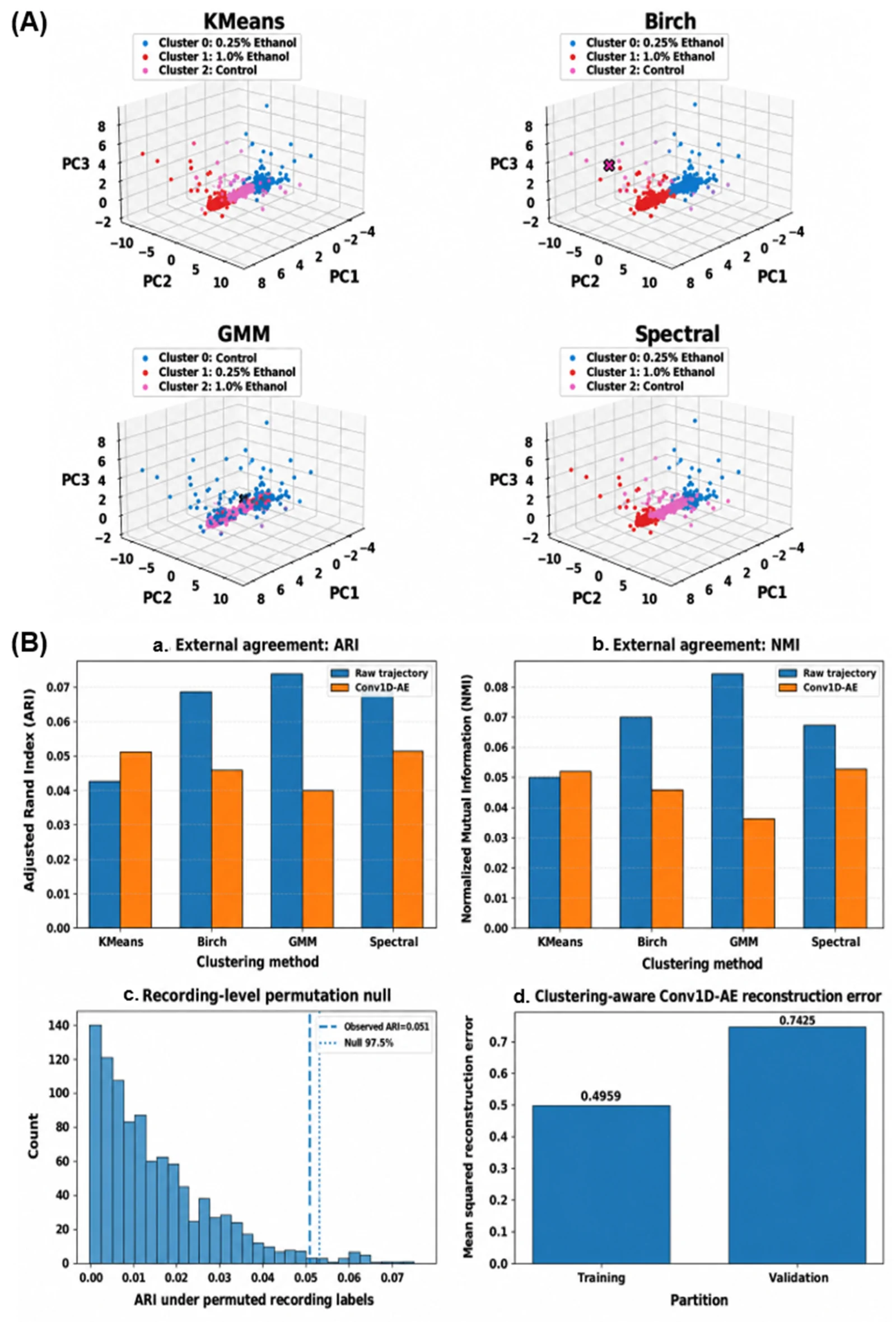
(**A**) The acute ethanol dataset after the autoencoder was applied using k = 3. Control and ethanol-treatment labels were used only for the comparison with respect to the predicted clusters. (**B**) (**a**) ARI and (**b**) MRI comparison for the control group, 0.25% ethanol, and 1% ethanol, respectively, during 5 min of the 3D locomotion test. (**c**) The recording-level permutation analysis shows the null distribution of ARI obtained by shuffling experimental labels at the recording level. (**d**) The reconstruction panel shows the mean squared reconstruction error for the training and held-out validation recordings.

To evaluate clustering performance on temporally ordered three-dimensional locomotor trajectories, five datasets representing interspecies differences, shoal sex composition, fin amputation, habituation time, and acute ethanol exposure were analyzed using K-Means, BIRCH, Gaussian Mixture Model (GMM), and Spectral Clustering. Continuous trajectory windows containing X, Y, and Z coordinates together with derived movement features were evaluated before and after autoencoder transformation. Internal clustering structure was assessed using the Silhouette score and Davies–Bouldin (DB) index, whereas external agreement with the predefined experimental groups was assessed using the ARI and NMI. Across the five datasets, the raw trajectory representation generally showed relatively low Silhouette scores and high DB index values, indicating limited internal cluster compactness and separation. After autoencoder transformation, Silhouette scores increased substantially for most dataset–algorithm combinations, while DB index values generally decreased, indicating improved internal clustering geometry (Table 1). The magnitude of improvement varied among datasets and clustering methods. In particular, the fin-amputation and ethanol datasets showed clear improvement after autoencoder transformation across both internal clustering metrics and external agreement measures, whereas the interspecies, shoal sex-composition, and habituation datasets showed stronger improvement in internal cluster compactness and separation than in ARI or NMI. Therefore, the autoencoder-derived latent representation generally enhanced the geometric organization of the trajectory data, although correspondence with the predefined experimental groups remained dataset- and method-dependent. The trajectory-level clustering results for each experimental dataset are presented in Figure 3, Figure 4, Figure 5, Figure 6 and Figure 7, together with the corresponding ARI and NMI comparisons. Detailed numerical results for the four clustering algorithms before and after autoencoder transformation are summarized in the associated tables while the detailed results of the inter-cluster separation evaluations can be found in Figure S1.

### 3.1. Clustering Results on the Interspecies Fish Dataset

First, the trajectory-level clustering framework was evaluated using locomotor data from zebrafish, medaka, and glass catfish. Continuous temporally ordered trajectory windows were analyzed using K-Means, BIRCH, GMM, and Spectral Clustering before and after autoencoder transformation. The 3D clustering results obtained from the autoencoder-based trajectory representation are shown in Figure 3, together with representative trajectories for the three species. Autoencoder transformation substantially improved the internal clustering structure across all four algorithms. After autoencoder transformation, Silhouette scores ranged from approximately 0.546 to 0.658, compared with approximately 0.079 to 0.132 for the raw trajectory representation (Table 2). Correspondingly, DB index values decreased from approximately 2.21–3.28 before autoencoder transformation to approximately 0.46–0.55 after autoencoder. This indicates substantially greater internal cluster compactness and separation. External agreement with the predefined species groups was additionally evaluated using the ARI and NMI, as shown in Figure 3B. ARI and NMI values indicated that the more compact latent clusters did not necessarily correspond directly to the predefined species labels. Therefore, the autoencoder primarily improved the geometric organization and separation of the trajectory representation, whereas correspondence with the experimental species groups remained dependent on the clustering algorithm.

### 3.2. Clustering Results on the Different Sexes in the Zebrafish Dataset

Second, the shoal sex-composition dataset, consisting of all-male, all-female, and mixed-sex groups at a 1:1 ratio, was analyzed using the same four clustering methods. The trajectory-level clustering results obtained after autoencoder transformation are presented in Figure 4 together with the corresponding external validation results. Autoencoder transformation significantly improved the internal clustering structure across all four algorithms. Silhouette scores increased from 0.140 to 0.547 for K-Means, from 0.083 to 0.671 for BIRCH, from 0.164 to 0.324 for GMM, and from 0.126 to 0.484 for Spectral Clustering (Table 3). Correspondingly, DB index values decreased from 2.273 to 0.795, 2.767 to 0.471, 2.709 to 2.252, and 2.282 to 0.857, respectively, indicating improved internal cluster compactness and separation. ARI values after autoencoder transformation were 0.075, 0.038, 0.107, and 0.099 for K-Means, BIRCH, GMM, and Spectral Clustering, respectively, while NMI values were 0.096, 0.083, 0.105, and 0.108. These results indicate that the autoencoder markedly improved the internal geometric organization of the clusters, whereas agreement with the predefined shoal sex-composition groups remained method-dependent.

### 3.3. Clustering Results on the Fin Amputation Effect in the Zebrafish Dataset

Third, the trajectory-based clustering analysis was applied to evaluate locomotor alterations following caudal-fin amputation, with and without lidocaine treatment. The four experimental groups were analyzed using 3D clustering results obtained after autoencoder transformation are presented in Figure 5. Autoencoder transformation markedly improved internal cluster compactness and separation across all four clustering algorithms. Silhouette scores increased from 0.132 to 0.618 for K-Means, from 0.111 to 0.576 for BIRCH, from 0.072 to 0.480 for GMM, and from 0.112 to 0.609 for Spectral Clustering (Table 4). Correspondingly, DB index values decreased from 1.951 to 0.708, 2.289 to 0.725, 2.710 to 1.985, and 2.009 to 0.665, respectively. In contrast to several other datasets, the fin-amputation dataset also showed improved external agreement after autoencoder transformation Figure 5. ARI increased from 0.033 to 0.087 for K-Means, from 0.050 to 0.095 for BIRCH, from 0.061 to 0.091 for GMM, and from 0.076 to 0.089 for Spectral Clustering. NMI likewise increased from 0.041 to 0.100, 0.053 to 0.109, 0.087 to 0.107, and 0.085 to 0.101, respectively. These findings indicate that the autoencoder-derived latent representation improved both internal clustering geometry and external validation with the predefined experimental groups in the caudal-fin-amputation dataset.

### 3.4. Clustering Results on the Different Time Observations During Zebrafish Habituation

Next, a clustering framework was applied to examine changes in zebrafish locomotor behavior during habituation to a novel environment. Fish behavior was recorded for 1 min at 5 min intervals up to 21 min of exposure, and the trajectory data from the five habituation time points were analyzed. The 3D clustering results obtained after autoencoder transformation are presented in Figure 6 together with the corresponding external validation results. Autoencoder transformation improved the internal clustering structure for all four algorithms, with Silhouette scores increasing from 0.136 to 0.455 for K-Means, from 0.101 to 0.337 for BIRCH, from 0.098 to 0.277 for GMM, and from 0.131 to 0.283 for Spectral Clustering (Table 5). The DB index decreased from 2.174 to 0.944 for K-Means, from 2.343 to 1.315 for BIRCH, and from 2.143 to 1.127 for Spectral Clustering, whereas GMM showed an increase from 2.611 to 3.106 after autoencoder transformation. These results indicate that the autoencoder generally improved internal cluster compactness and separation, although the magnitude of improvement was algorithm-dependent. External agreement with the predefined habituation time groups remained limited. ARI changed from 0.080 to 0.105 for K-Means, from 0.089 to 0.085 for BIRCH, from 0.080 to 0.065 for GMM, and from 0.071 to 0.086 for Spectral Clustering. NMI values changed from 0.120 to 0.111, 0.122 to 0.103, 0.113 to 0.091 and 0.113 to 0.106, respectively. Thus, although the autoencoder generally improved the geometric organization of the trajectory data, correspondence between the unsupervised clusters and the predefined habituation time points remained weak and method-dependent.

### 3.5. Clustering Results on the Acute Ethanol Effects on Zebrafish Behaviors Dataset

Finally, the performance of the current clustering framework was evaluated using zebrafish groups exposed to different ethanol concentrations. The 3D clustering results obtained after autoencoder transformation, with representative trajectories from each experimental group and shown in Figure 7. Based on the group-level clustering-quality analysis, Silhouette scores after autoencoder transformation ranged from 0.195 to 0.474, while DB index values ranged from 0.745 to 1.540 (Table 6). Compared with the original trajectory representation, the autoencoder generally increased Silhouette scores and reduced DB index values across most groups in algorithm combinations, indicating improved cluster compactness and separation. However, the magnitude of improvement varies among ethanol concentrations and clustering methods, with several small exceptions observed in the 1.0% ethanol group. Across all combinations after autoencoder transformation, the mean Silhouette score was 0.338 ± 0.085, while the mean DB index was 1.089 ± 0.259. These findings indicate that ethanol-associated trajectory patterns could be characterized by the clustering framework, although the degree of separation remained dependent on ethanol concentration and clustering algorithm. The detailed ARI comparison is also shown in Figure 7B.

### 3.6. PCA and Heatmap Clustering from the Manually Calculated Behavior Endpoints

**Figure 8:**
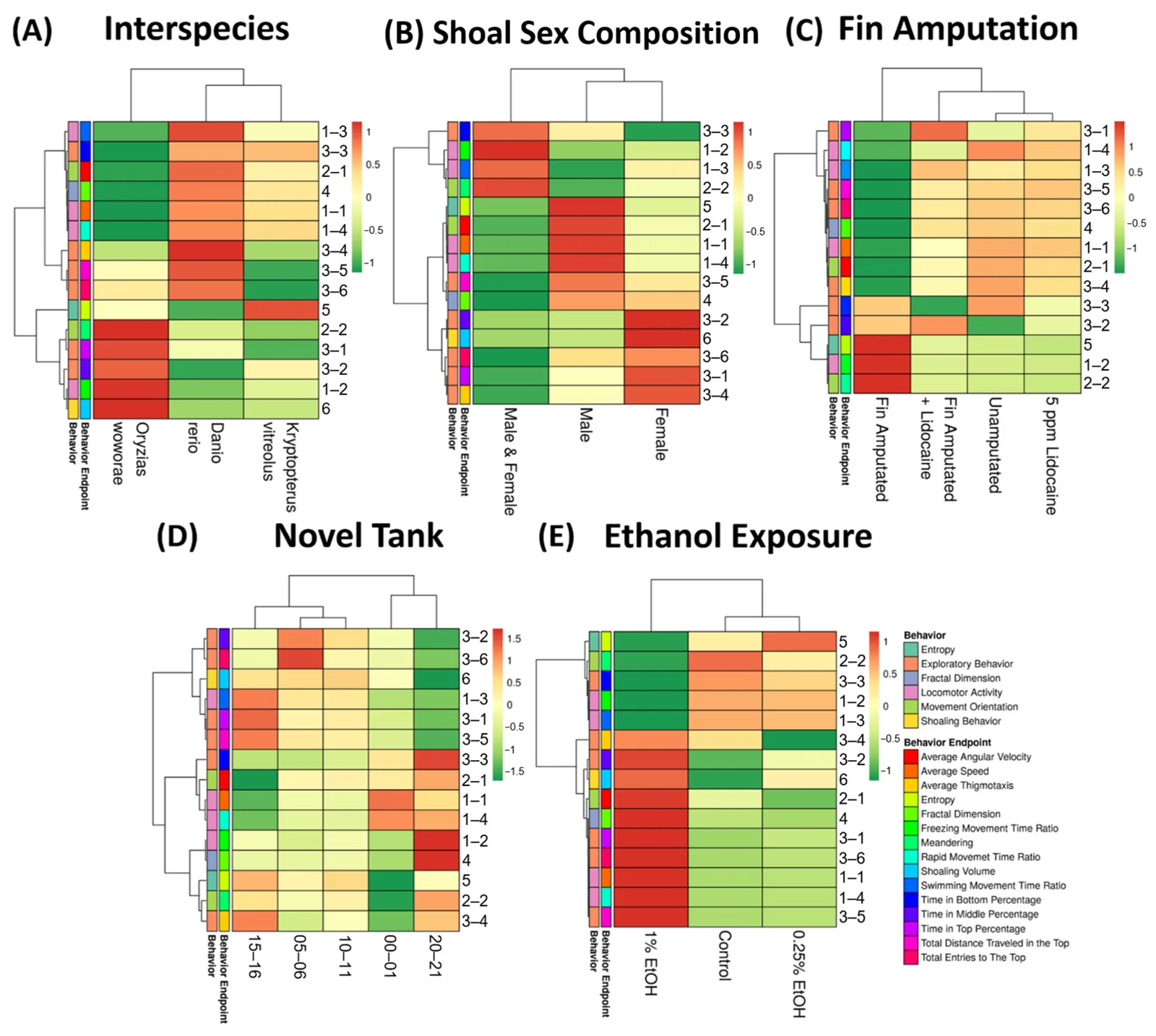
Hierarchical clustering analysis of multiple behavior endpoints in fish from (**A**) interspecies, (**B**) shoal sex composition effect, (**C**) caudal fin amputation, (**D**) novel tank exposure, and (**E**) acute ethanol exposure experiments. Detailed information regarding the behavior endpoints can be found in Table S1.

Finally, behavioral endpoint heatmaps were used as complementary qualitative references for biological interpretation of the unsupervised clustering patterns. Based on the first heatmap clustering result from the interspecies experiment, zebrafish showed relatively more similar behavior patterns to glass catfish (*K. vitreolus*), which displayed high similarity in most of the behavior categories, except for several behavior endpoints regarding the fish preference in the top portion of the tank. Meanwhile, medaka (*O. woworae*) showed more distinct behavior from those fish since it possessed lower locomotor activity and different exploratory behaviors (Figure 8A and Figure S2). Meanwhile, regarding the sex effect experiment result, each group displayed a relatively distinct heatmap pattern from the others, indicating that sex might play a role in the behavioral performance of zebrafish in this behavior test (Figure 8B and Figure S3). Next, as expected, the fin-amputated group possessed the most distinct behavioral pattern among other groups in the fin amputation experiment, which was then followed by the fin-amputated group that was treated with lidocaine, leaving the unamputated and lidocaine-treated groups in another cluster since both of them shared relatively similar behavior patterns (Figure 8C and Figure S4). Interestingly, in the novel tank experiment, the fish groups during the first and last minutes of the recordings were clustered together, leaving the other groups in another cluster. This result might indicate that the first minute of novel tank exposure was the habituation process for the fish, while the last minute represented their behavior after they had already acclimated to the test tank, thus leaving the habituation process that occurred in between in another cluster (Figure 8 and Figure S5). Finally, as expected, acute exposure to ethanol at the higher concentration (1%) was also found to alter fish behaviors by inducing hyperactivity-like behaviors in the exposed fish. Meanwhile, the lower dose of ethanol did not cause any significant effect on the overall zebrafish behaviors (Figure 8E and Figure S6).

### 3.7. GUI Operation

**Figure 9:**
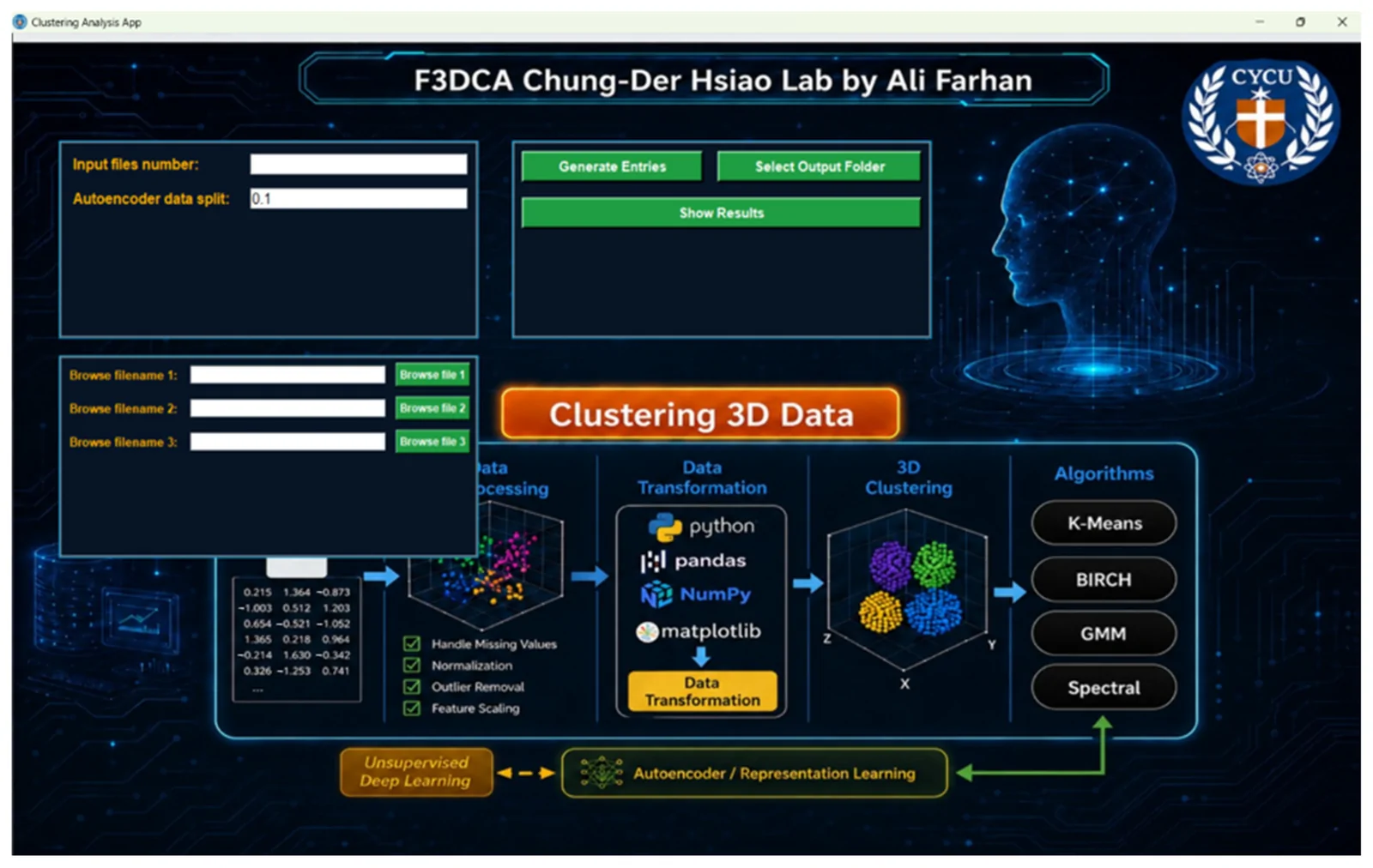
Layout of the designed GUI for the Fish 3D Clustering APP (F3DCA). The number of Excel files to be analyzed can be initially set by inserting the number in “Input files number”, followed by confirmation by clicking the “Generate Entries” button. The location of the output files can be chosen by clicking the “Select Output Folder” button. The “autoencoder data split” parameter allows the user to specify the proportion of data reserved for validation during the autoencoder model training. Finally, the Excel files can be loaded into the APP by clicking the “Browse file” button, followed by the “Show Results” button to execute the clustering process.

Finally, to offer a user-friendly application (APP), a Graphical User Interface (GUI) was also developed to reduce the complexity and laborious work of manual data calculation and to boost data calculation efficiency. Here, the APP was created using Python to provide easy installation, with a “start.bat” file for the execution of the APP installation process. Afterward, users can generate a number of entries according to the desired number of trajectory files in the form of X, Y, and Z trajectory data files. Subsequently, the APP would generate results using traditional clustering models as well as with the implementation of DL auto-encoder-based clustering to obtain higher separation power in each dataset. The user-friendly GUI layout can be seen in Figure 9.

## 4. Discussion

Behavior analysis is crucial in numerous real-world applications, including studying genetics, molecular biology, and cell biology. In zebrafish research, 3D trajectories are essential for accurately characterizing the behavior of this animal model organism. To the best of our knowledge, only a limited number of studies have applied unsupervised machine learning to animal behavioral datasets. Therefore, this study introduced an unsupervised machine learning framework to analyze 3D locomotion behavior in fish, with a particular focus on zebrafish. The clustering analysis was performed on their 3D trajectories using four different algorithms without relying on predefined behavioral endpoints. Based on the results, we found that the developed 3D trajectory analysis provided a useful approach to examine such complex behavioral responses in greater detail, as indicated by the clustering results, which were compared with conventional behavioral endpoints used as complementary qualitative information for biological interpretation. Instead of relying only on conventional behavioral endpoints, such as total distance or average speed, the proposed method evaluates the full spatial movement pattern of zebrafish. This allows differences in escape-related locomotion, stress-induced movement, and neuromuscular performance to be captured through trajectory-based clustering, providing a more comprehensive understanding of startle-related behavioral changes across different experimental conditions [52].

The analytical concept in the original clustering analysis was based on single frame-level XYZ coordinate observations. Because observations from the relevant experimental groups were pooled and standardized prior to clustering without maintaining temporal order, these initial clusters should primarily be interpreted as recurrent spatial occupancy and distribution patterns rather than temporal behavioral sequences. To address this limitation, the revised analysis incorporated temporally ordered trajectory windows containing both spatial and derived movement features, allowing the framework to characterize local locomotor dynamics rather than spatial occupancy alone. In the caudal-fin-amputation experiment, the amputated group showed a distinct locomotor pattern compared with the other experimental groups. However, this alteration should not be attributed exclusively to pain. Caudal-fin amputation may affect locomotion through multiple, potentially overlapping mechanisms, including nociceptive responses, procedural stress, and direct biomechanical impairment of propulsion and swimming performance. Therefore, the trajectory-based clustering results should be interpreted as evidence of altered locomotor organization following caudal-fin amputation, whereas identification of the specific biological mechanisms underlying these changes requires complementary physiological and behavioral measurements. For the acute ethanol experiment, the 1% ethanol-treated group displayed a more distinct trajectory pattern than the untreated and 0.25% ethanol groups (Figure 7). This clustering difference was interpreted together with the conventional behavioral endpoints derived from the same recordings. The high-dose group showed changes in locomotor measures consistent with increased activity, whereas the lower-dose group exhibited a more similar behavioral profile to the control group. Therefore, the clustering result indicates an ethanol-associated alteration in locomotor trajectory structure, while characterization of the specific behavioral response requires examination of interpretable variables such as swimming speed, movement-state duration, angular movement, and spatial exploration.

The current study consisted of two fundamental stages. The first stage entailed the application of four specific clustering methods (K-Means, BIRCH, GMM, and Spectral clustering) to the fish 3D trajectories dataset. Later, the clustering results were evaluated using Silhouette scores and the DB index. Silhouette score, one of the calculated scoring functions, according to Zhao et al.’s principle, indicates that any value within the range indicates good separation power by the clustering method [53,54]. Meanwhile, for the DB index, lower scores indicated better separation and more distinct clusters. However, after further evaluation of the fish 3D trajectories, it was found that the data structure of each group in every dataset varies due to the overlapping and differential topological significances; thus, this led to the second stage, in which a deep-learning autoencoder model was applied. The original trajectory features already showed some clustering structure, as indicated by the high DB index before autoencoder transformation. After autoencoder transformation, the DB index decreased, in some cases suggesting that the autoencoder improved the latent representation by reducing noise and enhancing cluster compactness and separation [55]. 1D CNNs have previously been applied to trajectory and movement data [56] because they can efficiently extract local patterns from ordered low-dimensional representations. Accordingly, a Conv1D architecture was adopted in the present autoencoder rather than a two-dimensional convolutional architecture, as the input consisted of compact coordinate vectors rather than image-like two-dimensional grids [42].

Next, the distinction between hierarchical and nonhierarchical (partitioning) approaches is necessary for a shared conceptual framework for cluster analysis and to support the current study’s features with behavioral impact; thus, inter-cluster and intra-cluster distances were analyzed. Inter-clustering evaluates the mean distance or difference among various clusters. The further distance between clusters, the more clearly defined and separated they are. Typically, the inter-cluster distance is computed by measuring the distance between the centroids or the minimum distance between data points in separate clusters [57]. In reference to statistics, it assesses how compact data points are in a cluster. The closer the data points are within the cluster, the more similar and tightly packed they are. The within-cluster distance is commonly measured by finding the average or maximum distance among all data points within a cluster. This analysis provided information on differences in the spatial organization and distribution of the fish trajectories across experimental groups. High-dimensional trajectory features may introduce noise and reduce clustering performance [58]. Therefore, an autoencoder-based framework was applied to learn a nonlinear 3D latent representation of the trajectory data before clustering. After autoencoder deployment, K-Means, BIRCH, and Spectral Clustering generally showed higher Silhouette scores than GMM across the datasets, indicating better cluster separation. The dendrogram-based inter-clustering analysis also showed clear relationships among these clustering methods after feature transformation. In the interspecies dataset, clustering quality differed among species and methods, with no single species consistently showing the highest Silhouette and lowest DB index values across all four methods. A similar trend was observed in the caudal fin-amputated group. In contrast, the 1% ethanol-treatment group and the habituation time-point groups showed internal clustering structure variations among datasets and algorithms. Autoencoder transformation generally increased Silhouette scores and reduced DB index values, indicating improved internal geometric organization. However, the magnitude of improvement was dataset- and method-dependent. In the sex-difference dataset, the clustering scores were comparable between groups, suggesting less distinct trajectory patterns. Although the autoencoder transformation improved clustering performance in most datasets, some groups showed limited improvement. This may be due to overlapping locomotion [59] patterns among fish groups or insufficient separation of similar trajectory features in the latent space.

Additionally, in comparison with the supervised method, which was represented as the behavioral endpoints calculation, the current unsupervised clustering method showed positive results. In the first experiment regarding the interspecies behavior differences, zebrafish were clustered together with glass catfish (*K. vitreolus*), indicating that zebrafish possessed relatively more different behavior than medaka (*O. woworae*) than glass catfish, which was also reflected in the behavior endpoints calculation results (Figure S2). This result is consistent with the previous study that demonstrated the difference in the behavior pattern between zebrafish and medaka (*O. woworae*) in various behavior tests, including novel tank, mirror biting, and circadian locomotor activity assays [60]. However, previous studies have applied machine-learning and clustering approaches to zebrafish 3D trajectory data, including the FuzzyART-based framework of Yang et al. (2021) [61]. The present study extends this area by integrating four unsupervised clustering algorithms with an autoencoder-based deep learning comparison for multiple experimental datasets. In addition, the present work includes an interspecies comparison involving zebrafish, medaka, and glass catfish. This combination has received limited attention in previous 3D behavioral studies. Thus, while some pattern differences are still clearly shown in the heatmap, both species showed more similar behaviors compared to *O. woworae*, which, interestingly, a prior study indicated a similar color preference ranking of *K. vitreolus* with *D. rerio* [62].

Nevertheless, the clustering scores by several clustering methods after the autoencoder indicated a separation between these three fish species that was also shown in the heatmap clustering result, which might be due to the relatively similar heatmap pattern in some behavior endpoints between zebrafish and glass catfish, despite the behavior differences of medaka from these species. Consequently, regarding the sex differences in affecting a group of zebrafish’s swimming performance, the results indicated that each group displayed a relatively distinct behavior pattern from the others, as shown in the heatmap clustering. These results are quite consistent with prior studies that demonstrated sex differences in zebrafish behavioral responses. As also shown in the current results, the adult all-male shoals test exhibited higher locomotion than the female group, while female fish displayed more robust exploratory behaviors that might indicate a less anxious response during the open field test tank (Figure S3). After a deeper investigation, this phenomenon may be related to the multiple gene transcripts that are differentially expressed in their brain and their gene ontology pathways [63,64]. Meanwhile, in the fin amputation experiment, as we expected, the fin-amputated group displayed the most distinct heatmap pattern compared to other groups, as also shown in the behavior endpoints calculation results (Figure S4). This behavioral alteration may reflect multiple consequences of caudal fin amputation, including nociceptive responses, procedural stress, and biomechanical impairment of swimming performance, considering the pain potency of this procedure in zebrafish that was demonstrated in previous studies, which caused a significant reduction in activity levels and movement complexity in the amputated fish [30]. As expected, since only one group showed a significantly different heatmap pattern from the other groups, the calculated Silhouette score did not indicate a relatively high separation power of the current clustering method in analyzing the data from these groups, which is in line with the DB index score that showed relatively higher values, as expected. Next, in evaluating the fish response toward a novel environment, we found that there are two major clusters, with the first and last observation groups in one cluster and the other groups in another cluster. This result is plausible since during the initial period of exposure to a new environment, zebrafish exhibit robust behavioral responses to anxiety evoked by novelty, and after several minutes, fish gradually increase in exploration, which was also observed in the behavior endpoints calculation results (Figure S5).

Finally, after the fish are acclimated to the environment, they will show a natural swimming behavior that could be indicated by the normal movement complexity [65,66]. Interestingly, the current unsupervised clustering method showed a moderate separation power in analyzing these groups. Based on the heatmap clustering results, we hypothesized that these results might be due to the majority of the groups (05–06, 10–11, and 15–16) possessing a relatively similar heatmap pattern that might reduce the difference values of these groups compared with other groups, although the other groups (00–01 and 20–21) displayed a relatively distinct overall heatmap pattern. Finally, in the heatmap clustering result from the last experiment, which was regarding acute ethanol exposure, the most distinct behavior pattern was exhibited by the high-dose group, while the low-dose group displayed a comparable heatmap pattern to the untreated fish. These results are consistent with the prior study that found acute exposure to ethanol at 1% concentration could increase the total distance moved by the fish, while the lower dose (0.5%) did not show a significant difference in locomotion compared to the control group, as shown in the behavior endpoints calculation results (Figure S6) [67]. Similar to the sex effect results above, both the Silhouette score and DB index for these data indicated a better separation power, which might be due to the significant differences in the swimming pattern of the 1% EtOH-treated group, despite the similarity of the heatmap pattern between the untreated and 0.25% EtOH-treated groups.

### Potential Limitations of the Study

Despite the advantages of the current study, it still faces several limitations. First, the clustering approach showed reduced separation power when different experimental groups displayed highly similar locomotion patterns. Because the unsupervised clustering results and conventional behavioral endpoints were derived from the same underlying trajectory recordings, agreement between these analyses should not be interpreted as independent biological or mechanistic validation. Instead, conventional endpoints provide complementary, interpretable information that contextualizes the spatial trajectory patterns identified by clustering. Consequently, this comparison assesses consistency between two analytical representations of the same locomotor data rather than establishing independent ground-truth validation. Second, although the method can identify whether clustering differences exist, it does not directly explain which specific behavioral endpoints, such as swimming speed, distance traveled, turning angle, or vertical position, are responsible for the observed differences. Therefore, conventional behavioral endpoint analysis remains necessary for biological interpretation of the clustering results. This suggests that unsupervised clustering may be less effective when behavioral differences are subtle or when trajectory features overlap substantially between groups. An additional limitation concerns uncertainty introduced by upstream multi-fish tracking. The six fish were tracked simultaneously, and the 3D coordinates were reconstructed from direct and mirrored views; temporary tracking loss, correspondence errors, or identity switches could introduce error in coordinate observations. Such errors could increase within-cluster dispersion or alter between-group separation and therefore affect Silhouette, Davies–Bouldin, ARI, and NMI values. The present clustering results should therefore be interpreted as conditional on the accuracy of the upstream tracking and 3D coordinate reconstruction. Future studies should retain frame-level tracking-confidence information and evaluate clustering robustness after controlled perturbation or removal of uncertain trajectory segments.

## 5. Conclusions

In conclusion, this study successfully developed an unsupervised 3D locomotion trajectory analysis software package for zebrafish behavioral assessment. The proposed unsupervised framework can serve as an automated screening tool for identifying differences in fish locomotor patterns. Autoencoder-based feature representation generally improved internal cluster separation in most datasets, while quantitative external agreement (ARI and NMI) with predefined experimental groups varied depending on the dataset and algorithm applied. Rather than serving as an independent quantitative validation, comparison with conventional behavioral endpoints provided qualitative, complementary information for interpreting the observed trajectory patterns. Because both analyses originate from the same tracking data, their comparison represents an evaluation between analytical approaches rather than independent biological validation. Therefore, the proposed method should complement, rather than replace, conventional behavioral analysis, particularly when detailed mechanistic interpretation is required. Overall, the proposed unsupervised clustering framework serves as an efficient, unbiased screening tool for identifying global 3D trajectory alterations in fish behavioral assays, offering a high-throughput precursor to detailed conventional endpoint analyses in various research fields.

## Figures and Tables

**Table 1:** Comparison of Silhouette and DB index scores for four clustering methods before applying a deep learning autoencoder. Sil: Silhouette; DB: Davies–Bouldin Index.

Dataset	Method	Raw Sil.	AE Sil.	Raw DB	AE DB	Raw ARI	AE ARI	Raw NMI	AE NMI
Interspecies	K-Means	0.132	0.658	2.209	0.477	0.043	0.054	0.050	0.056
	BIRCH	0.091	0.645	2.498	0.489	0.051	0.050	0.055	0.054
	GMM	0.079	0.546	3.283	0.548	0.163	0.036	0.203	0.041
	Spectral	0.125	0.656	2.247	0.460	0.046	0.053	0.058	0.054
Shoal sex	K-Means	0.140	0.547	2.273	0.795	0.124	0.075	0.123	0.096
	BIRCH	0.083	0.671	2.767	0.471	0.053	0.038	0.071	0.083
	GMM	0.164	0.324	2.709	2.252	0.117	0.107	0.171	0.105
	Spectral	0.126	0.484	2.282	0.857	0.114	0.099	0.108	0.108
Fin amputation	K-Means	0.132	0.618	1.951	0.708	0.033	0.087	0.041	0.100
	BIRCH	0.111	0.576	2.289	0.725	0.050	0.095	0.053	0.109
	GMM	0.072	0.480	2.710	1.985	0.061	0.091	0.087	0.107
	Spectral	0.112	0.609	2.009	0.665	0.076	0.089	0.085	0.101
Habituation	K-Means	0.136	0.455	2.174	0.944	0.080	0.105	0.120	0.111
	BIRCH	0.101	0.337	2.343	1.315	0.089	0.085	0.122	0.103
	GMM	0.098	0.277	2.611	3.106	0.080	0.065	0.113	0.091
	Spectral	0.131	0.283	2.143	1.127	0.071	0.086	0.113	0.106
Acute ethanol	K-Means	0.115	0.566	2.098	0.583	0.042	0.186	0.058	0.245
	BIRCH	0.077	0.483	2.212	0.678	0.117	0.187	0.156	0.236
	GMM	0.111	0.376	2.082	1.290	0.175	0.208	0.197	0.228
	Spectral	0.084	0.553	2.103	0.595	0.087	0.178	0.177	0.249

**Table 2:** Comparison of Silhouette and DB index scores of the interspecies fish behavior dataset calculated by four clustering methods before and after applying a deep learning autoencoder.

Group	Method	Silhouette Before	Silhouette After	DB Before	DB After
Interspecies	K-Means	0.132	0.658	2.209	0.477
Interspecies	BIRCH	0.091	0.645	2.498	0.489
Interspecies	GMM	0.079	0.546	3.283	0.548
Interspecies	Spectral	0.125	0.656	2.247	0.460

**Table 3:** Comparison of Silhouette and DB index scores of different sexes on the zebrafish behaviors dataset, calculated by four clustering methods before and after applying a deep learning autoencoder.

Group	Method	Sil. Before	Sil. After	DB Before	DB After
Male–Male	K-Means	0.349	0.414	1.126	0.867
Male–Male	BIRCH	0.324	0.377	1.158	0.914
Male–Male	GMM	0.231	0.313	1.706	1.208
Male–Male	Spectral	0.337	0.401	1.124	0.890
Female–Female	K-Means	0.337	0.379	1.147	1.003
Female–Female	BIRCH	0.300	0.307	1.269	1.155
Female–Female	GMM	0.220	0.292	1.340	1.095
Female–Female	Spectral	0.324	0.375	1.189	0.992
Male–Female	K-Means	0.341	0.390	1.161	0.872
Male–Female	BIRCH	0.288	0.327	1.330	1.037
Male–Female	GMM	0.212	0.175	1.808	2.573
Male–Female	Spectral	0.322	0.378	1.090	0.927

**Table 4:** Comparison of Silhouette and DB index scores of the caudal fin amputation effect on zebrafish behaviors dataset calculated by four clustering methods before and after applying a deep learning autoencoder.

Group	Method	Sil. Before	Sil. After	DB Before	DB After
Unamputated	K-Means	0.123	0.623	2.285	0.780
Unamputated	BIRCH	0.075	0.514	2.446	1.064
Unamputated	GMM	0.061	0.420	2.637	2.093
Unamputated	Spectral	0.072	0.632	2.209	0.713
Caudal Fin Amputated	K-Means	0.118	0.515	1.887	0.646
Caudal Fin Amputated	BIRCH	0.097	0.574	2.400	0.506
Caudal Fin Amputated	GMM	−0.009	0.409	3.102	2.825
Caudal Fin Amputated	Spectral	0.101	0.482	1.953	0.617
Caudal Fin Amputated + Lidocaine	K-Means	0.142	0.653	1.813	0.659
Caudal Fin Amputated + Lidocaine	BIRCH	0.107	0.542	2.106	0.918
Caudal Fin Amputated + Lidocaine	GMM	0.064	0.536	2.723	2.318
Caudal Fin Amputated + Lidocaine	Spectral	0.124	0.634	1.830	0.671
Lidocaine only	K-Means	0.153	0.669	1.739	0.675
Lidocaine only	BIRCH	0.150	0.570	1.964	0.705
Lidocaine only	GMM	0.100	0.491	2.255	2.767
Lidocaine only	Spectral	0.149	0.672	1.722	0.623

**Table 5:** Comparison of Silhouette and DB index scores of different observation times during the zebrafish habituation process behaviors dataset, calculated by four clustering methods before and after applying a deep learning autoencoder.

Time Group	Method	Sil. Before	Sil. After	DB Before	DB After
0–1 min	K-Means	0.338	0.330	0.967	1.039
0–1 min	BIRCH	0.290	0.309	1.069	1.057
0–1 min	GMM	0.107	0.179	1.598	1.372
0–1 min	Spectral	0.320	0.284	0.981	1.057
5–6 min	K-Means	0.390	0.377	0.879	0.916
5–6 min	BIRCH	0.377	0.335	0.941	1.073
5–6 min	GMM	0.053	0.230	1.766	1.222
5–6 min	Spectral	0.377	0.389	0.880	0.834
10–11 min	K-Means	0.387	0.366	0.922	0.934
10–11 min	BIRCH	0.347	0.344	1.010	1.039
10–11 min	GMM	0.216	0.256	1.225	1.333
10–11 min	Spectral	0.374	0.307	0.872	1.026
15–16 min	K-Means	0.372	0.385	0.873	0.931
15–16 min	BIRCH	0.321	0.324	0.932	1.145
15–16 min	GMM	0.152	0.281	1.370	1.138
15–16 min	Spectral	0.372	0.337	0.779	1.012
20–21 min	K-Means	0.377	0.414	0.837	0.834
20–21 min	BIRCH	0.335	0.380	0.910	0.840
20–21 min	GMM	0.148	0.388	1.973	0.921
20–21 min	Spectral	0.338	0.381	0.838	0.803

**Table 6:** Comparison of Silhouette and DB index scores of acute ethanol exposure effect on zebrafish behaviors dataset calculated by four clustering methods before and after applying deep learning autoencoder.

Group	Method	Silhouette Before	Silhouette After	DB Before	DB After
Control	K-Means	0.298	0.348	1.191	1.063
Control	BIRCH	0.252	0.297	1.439	1.261
Control	GMM	0.158	0.230	1.688	1.428
Control	Spectral	0.299	0.352	1.244	1.006
0.25% Ethanol	K-Means	0.403	0.474	0.888	0.745
0.25% Ethanol	BIRCH	0.353	0.405	0.993	0.820
0.25% Ethanol	GMM	0.301	0.366	1.392	0.873
0.25% Ethanol	Spectral	0.397	0.464	0.881	0.760
1.0% Ethanol	K-Means	0.299	0.304	1.177	1.213
1.0% Ethanol	BIRCH	0.284	0.304	1.206	1.223
1.0% Ethanol	GMM	0.157	0.195	1.612	1.540
1.0% Ethanol	Spectral	0.308	0.297	1.143	1.139

## Data Availability

All of the trajectory data for each dataset used in the current study are available on Zenodo (https://doi.org/10.5281/zenodo.14964942). The developed APP, including its source code, is available on GitHub at https://github.com/Alizebrafish/3D_Clustering (accessed on 23 September 2026). The version of source code used to generate the primary analyses, Figure 3, Figure 4, Figure 5, Figure 6 and Figure 7 and Table 1, Table 2, Table 3, Table 4, Table 5 and Table 6, is provided in the accompanying software package as the primary analysis of the original release. The package includes the original analysis script, parameter settings, dependency requirements, and instructions for the reported analyses. The supplementary ARI/NMI external-validation workflow is provided separately as supplementary validation v2. The version identifiers and associated documentation are available on the respective repository on GitHub.

## Supplementary Materials

The following supporting information can be downloaded at https://www.mdpi.com/article/10.3390/biology15191768/s1, Table S1. Summary of the fish behavioral endpoints measured in each experiment. Figure S1. Dendrograms based on Silhouette scores and Davies–Bouldin index values obtained after autoencoder transformation for the four clustering methods. Panels show (A,B) interspecies comparison, (C,D) shoal sex composition, (E,F) caudal-fin amputation, (G,H) habituation time, and (I,J) acute ethanol exposure. For each dataset, the left panel represents clustering relationships based on Silhouette-score profiles and the right panel represents relationships based on Davies–Bouldin-index profiles. Figure S2. Comparison of behavior endpoints between *Danio rerio*, *Oryzias woworae*, and *Kryptopterus vitreolus* in the 3D locomotion test (shoal size = six fish). The calculated (A) average speed, (B) freezing, (C) swimming, and (D) rapid movement time ratios indicated fish locomotion, while (E) average angular velocity and (F) meandering belong to the movement orientation endpoints group. Furthermore, to evaluate fish exploratory behaviors, (G) average distance to the center of the tank, (H) total distance traveled at the top, (I) number of entries to the top, and time in the (J) top, (K) middle, and (L) bottom durations were also calculated. Finally, (M) fractal dimension, (N) entropy, and (O) shoaling volume were also calculated. Data are expressed as median with 95% CI and were analyzed by Nested one-way ANOVA followed by Tukey’s multiple comparisons test, except (O), which was analyzed by Kruskal–Wallis test followed by Dunn’s multiple comparisons test. Different letters signify a statistical difference (*p* < 0.05; *n* = 3 (18 fish)). Figure S3. Comparison of behavior endpoints among all-male, all-female, and mixed-sex zebrafish shoals (shoal size = six fish). The calculated (A) average speed, (B) freezing, (C) swimming, and (D) rapid movement time ratios indicated fish locomotion, while (E) average angular velocity and (F) meandering belong to the movement orientation endpoints group. Furthermore, to evaluate fish exploratory behaviors, (G) average distance to the center of the tank, (H) total distance traveled at the top, (I) number of entries to the top, and time in the (J) top, (K) middle, and (L) bottom durations were also calculated. Finally, (M) fractal dimension, (N) entropy, and (O) shoaling volume were also calculated. Data are expressed as median with 95% CI and were analyzed by Nested one-way ANOVA followed by Tukey’s multiple comparisons test, except (O), which was analyzed by Kruskal–Wallis test followed by Dunn’s multiple comparisons test. Different letters signify a statistical difference (*p* < 0.05; *n* = 3 (18 fish)). Figure S4. Comparison of behavior endpoints between unamputated, caudal fin-amputated, caudal fin-amputated with 5 ppm lidocaine, and 5 ppm lidocaine-treated zebrafish in the 3D locomotion test at 1 h post-amputation (shoal size = six fish). The calculated (A) average speed, (B) freezing, (C) swimming, and (D) rapid movement time ratios indicated fish locomotion, while (E) average angular velocity and (F) meandering represented movement-orientation endpoints. Furthermore, to evaluate exploratory behavior, (G) average distance to the center of the tank, (H) total distance traveled at the top, (I) number of entries to the top, and time spent in the (J) top, (K) middle, and (L) bottom zones were calculated. Finally, (M) fractal dimension, (N) entropy, and (O) shoaling volume were calculated. Data are expressed as median with 95% CI and were analyzed by Nested one-way ANOVA followed by Tukey’s multiple-comparisons test. Different letters indicate a statistically significant difference (*p* < 0.05; *n* = 3 shoals, 18 fish). Figure S5. Comparison of behavior endpoints from a 1 min observation time between 5 min time intervals of zebrafish (shoal size = six fish). The calculated (A) average speed, (B) freezing, (C) swimming, and (D) rapid movement time ratios indicated fish locomotion, while (E) average angular velocity and (F) meandering belong to the movement orientation endpoints group. Furthermore, to evaluate fish exploratory behaviors, (G) average distance to the center of the tank, (H) total distance traveled at the top, (I) number of entries to the top, and time in the (J) top, (K) middle, and (L) bottom durations were also calculated. Finally, (M) fractal dimension, (N) entropy, and (O) shoaling volume were also calculated. Data are expressed as median with 95% CI and were analyzed by Nested one-way ANOVA followed by Tukey’s multiple comparisons test, except (O), which was analyzed by Kruskal–Wallis test followed by Dunn’s multiple comparisons test (*n* = 3 (18 fish)). Figure S6. Comparison of behavior endpoints between zebrafish acutely exposed to 0% (control), 0.25%, and 1% ethanol in the 3D locomotion test (shoal size = six fish). Control: 18 fish = 3 shoals, and each ethanol treatment: 12 fish = 2 shoals. The calculated (**A**) average speed, (**B**) freezing, (**C**) swimming, and (**D**) rapid movement time ratios indicated fish locomotion, while (**E**) average angular velocity and (**F**) meandering belong to the movement orientation endpoints group. Furthermore, to evaluate fish exploratory behaviors, (**G**) average distance to the center of the tank, (**H**) total distance traveled at the top, (**I**) number of entries to the top, and time in the (**J**) top, (**K**) middle, and (**L**) bottom durations were also calculated. Finally, (**M**) fractal dimension, (**N**) entropy, and (**O**) shoaling volume were also calculated. Data are expressed as median with 95% CI and were analyzed by Nested one-way ANOVA followed by Tukey’s multiple comparisons test, except (**O**), which was analyzed by Kruskal–Wallis test followed by Dunn’s multiple comparisons test. Different letters signify a statistical difference (*p* < 0.05; *n* control = 3 (18 fish), *n* EtOH treatment = 2 (12 fish)).
