# Supplementary material for: Fish 3D Clustering App (F3DCA): A Non-Supervised Clustering Method with a Machine Learning Autoencoder for Analyzing 3D Trajectories of Three Fish Species
Source: Biology (Basel). 2026 Oct 4;15(19):1768. doi: 10.3390/biology15191768 (PMC13664162; doi:10.3390/biology15191768)
Supplement: Supplementary file 1 [file biology-15-01768-s001.zip › Figure S1.pdf]

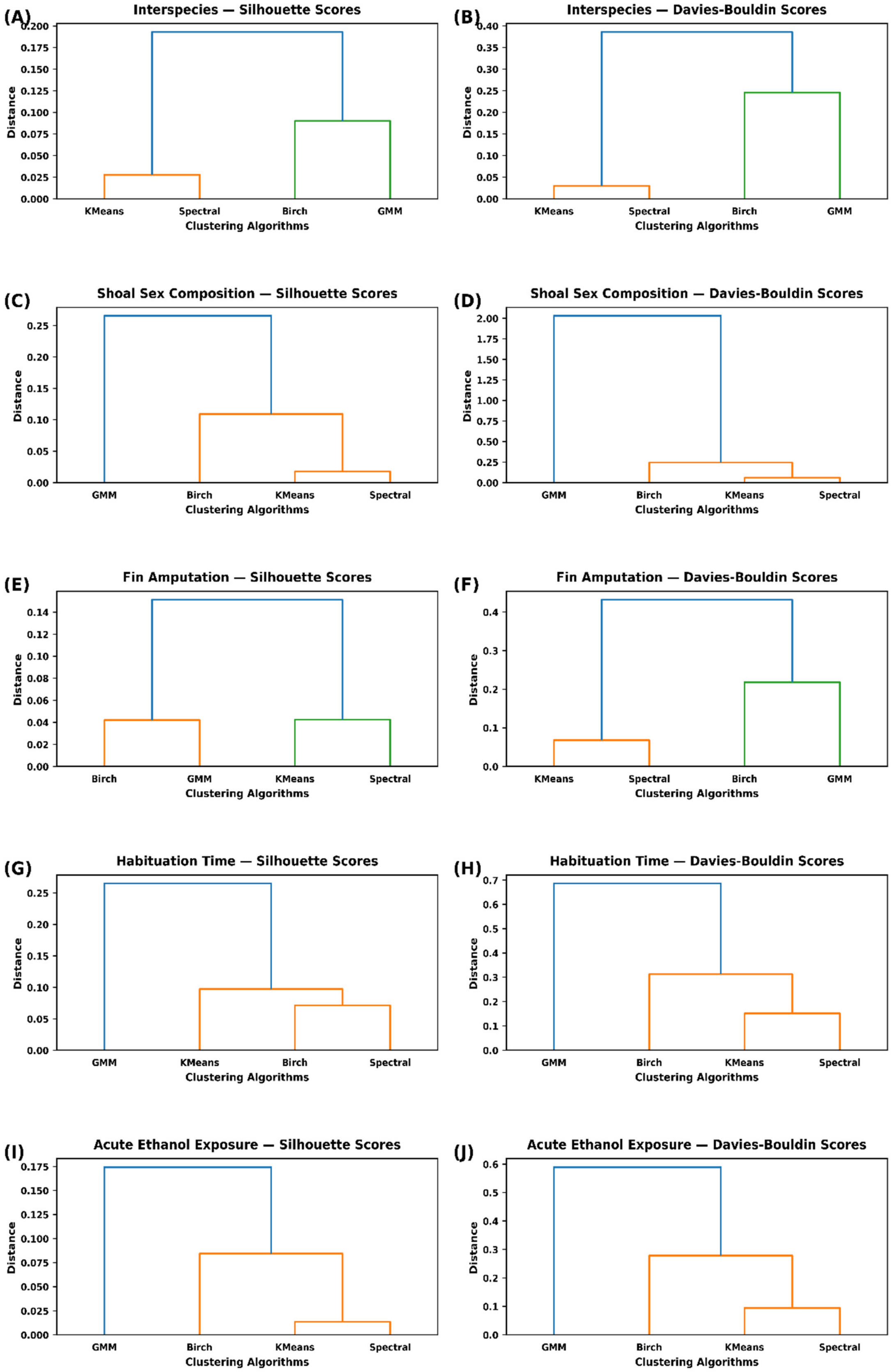
(A)
Interspecies — Silhouette Scores
(B)
Interspecies — Davies-Bouldin Scores
(C)
Shoal Sex Composition — Silhouette Scores
(D)
Shoal Sex Composition — Davies-Bouldin Scores
(E)
Fin Amputation — Silhouette Scores
(F)
Fin Amputation — Davies-Bouldin Scores
(G)
Habituation Time — Silhouette Scores
(H)
Habituation Time — Davies-Bouldin Scores
(I)
Acute Ethanol Exposure — Silhouette Scores
(J)
Acute Ethanol Exposure — Davies-Bouldin Scores
Distance
Clustering Algorithms
KMeans
Spectral
Birch
GMM

Figure S1. Dendrograms based on Silhouette scores and Davies–Bouldin index values obtained after autoencoder transformation for the four clustering methods. Panels show **(A,B)** interspecies comparison, **(C,D)** shoal sex composition, **(E,F)** caudal-fin amputation, **(G,H)** habituation time, and **(I,J)** acute ethanol exposure. For each dataset, the left panel represents clustering relationships based on Silhouette-score profiles and the right panel represents relationships based on Davies–Bouldin-index profiles.
