# Supplementary material for: Fish 3D Clustering App (F3DCA): A Non-Supervised Clustering Method with a Machine Learning Autoencoder for Analyzing 3D Trajectories of Three Fish Species
Source: Biology (Basel). 2026 Oct 4;15(19):1768. doi: 10.3390/biology15191768 (PMC13664162; doi:10.3390/biology15191768)
Supplement: Supplementary file 1 [file biology-15-01768-s001.zip › Figure S3.pdf]

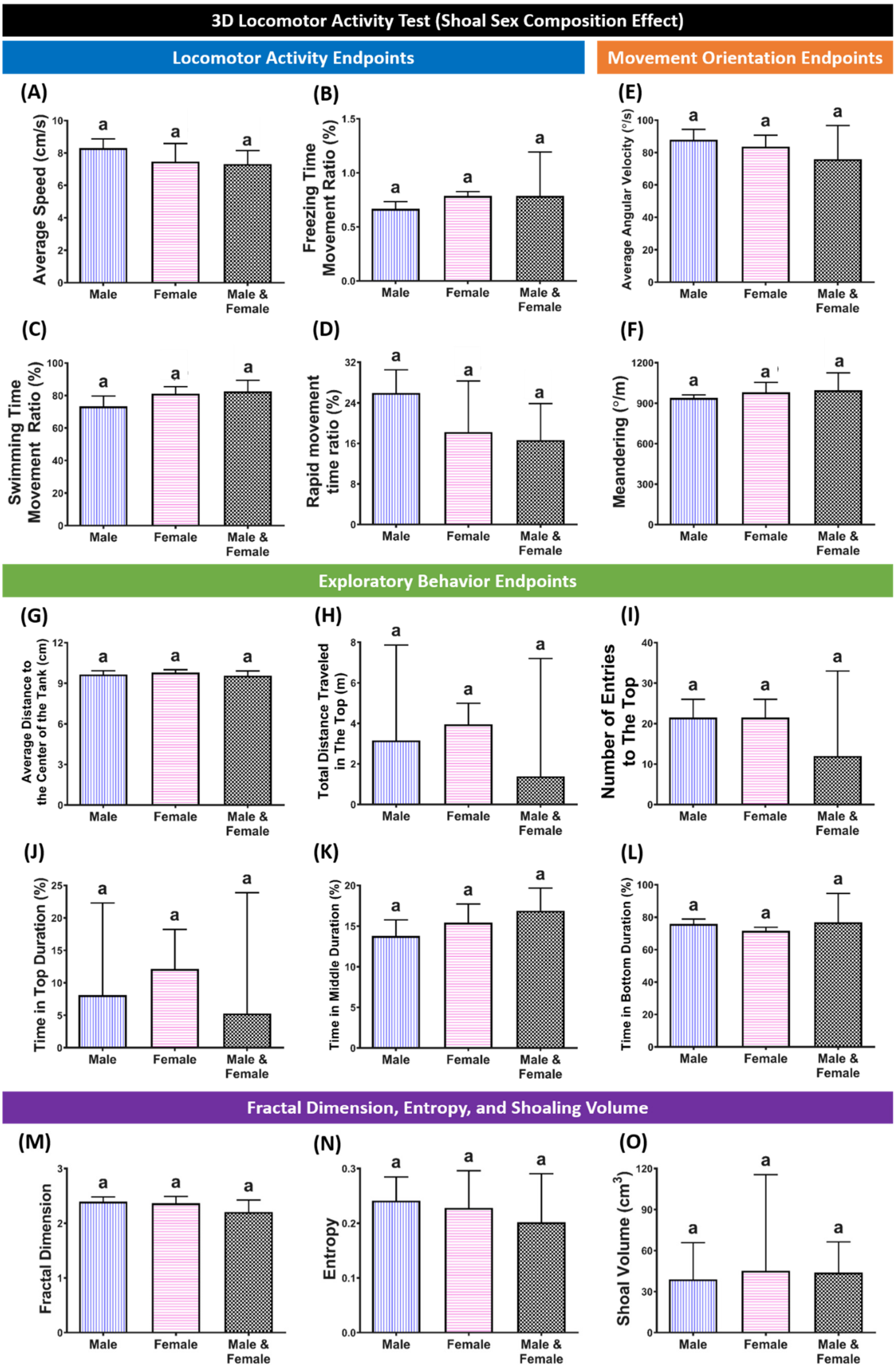
3D Locomotor Activity Test (Shoal Sex Composition Effect)
Locomotor Activity Endpoints
Movement Orientation Endpoints
(A)
Average Speed (cm/s)
(B)
Freezing Time Movement Ratio (%)
(E)
Average Angular Velocity (°/s)
(C)
Swimming Time Movement Ratio (%)
(D)
Rapid movement time ratio (%)
(F)
Meandering (°/m)
Exploratory Behavior Endpoints
(G)
Average Distance to the Center of the Tank (cm)
(H)
Total Distance Traveled in The Top (m)
(I)
Number of Entries to The Top
(J)
Time in Top Duration (%)
(K)
Time in Middle Duration (%)
(L)
Time in Bottom Duration (%)
Fractal Dimension, Entropy, and Shoaling Volume
(M)
Fractal Dimension
(N)
Entropy
(O)
Shoal Volume (cm³)
Male
Female
Male & Female
a

Figure S3. Comparison of behavior endpoints between gender combinations of zebrafish in the 3D locomotion test (shoal size = six fish). The calculated **(A)** average speed, **(B)** freezing, **(C)** swimming, and **(D)** rapid movement time ratios indicated the fish locomotion while **(E)** average angular velocity and **(F)** meandering belong to the movement orientation endpoints group. Furthermore, to evaluate fish exploratory behaviors, **(G)** average distance to the center of the tank, **(H)** total distance traveled at the top, **(I)** number of entries to the top, time in the **(J)** top, **(K)** middle, and **(L)** bottom durations, were also calculated. Finally, **(M)** fractal dimension, **(N)** entropy, and **(O)** shoaling volume were also calculated. Data are expressed as median with 95% CI and were analyzed by Nested one-way ANOVA followed by Tukey's multiple comparisons test, except (O), which was analyzed by Kruskal-Wallis test followed by Dunn's multiple comparisons test. Different letters signify a statistical difference ($P < 0.05$; $n = 3$ (18 fish)).
