# Supplementary material for: Fish 3D Clustering App (F3DCA): A Non-Supervised Clustering Method with a Machine Learning Autoencoder for Analyzing 3D Trajectories of Three Fish Species
Source: Biology (Basel). 2026 Oct 4;15(19):1768. doi: 10.3390/biology15191768 (PMC13664162; doi:10.3390/biology15191768)
Supplement: Supplementary file 1 [file biology-15-01768-s001.zip › Figure S4.pdf]

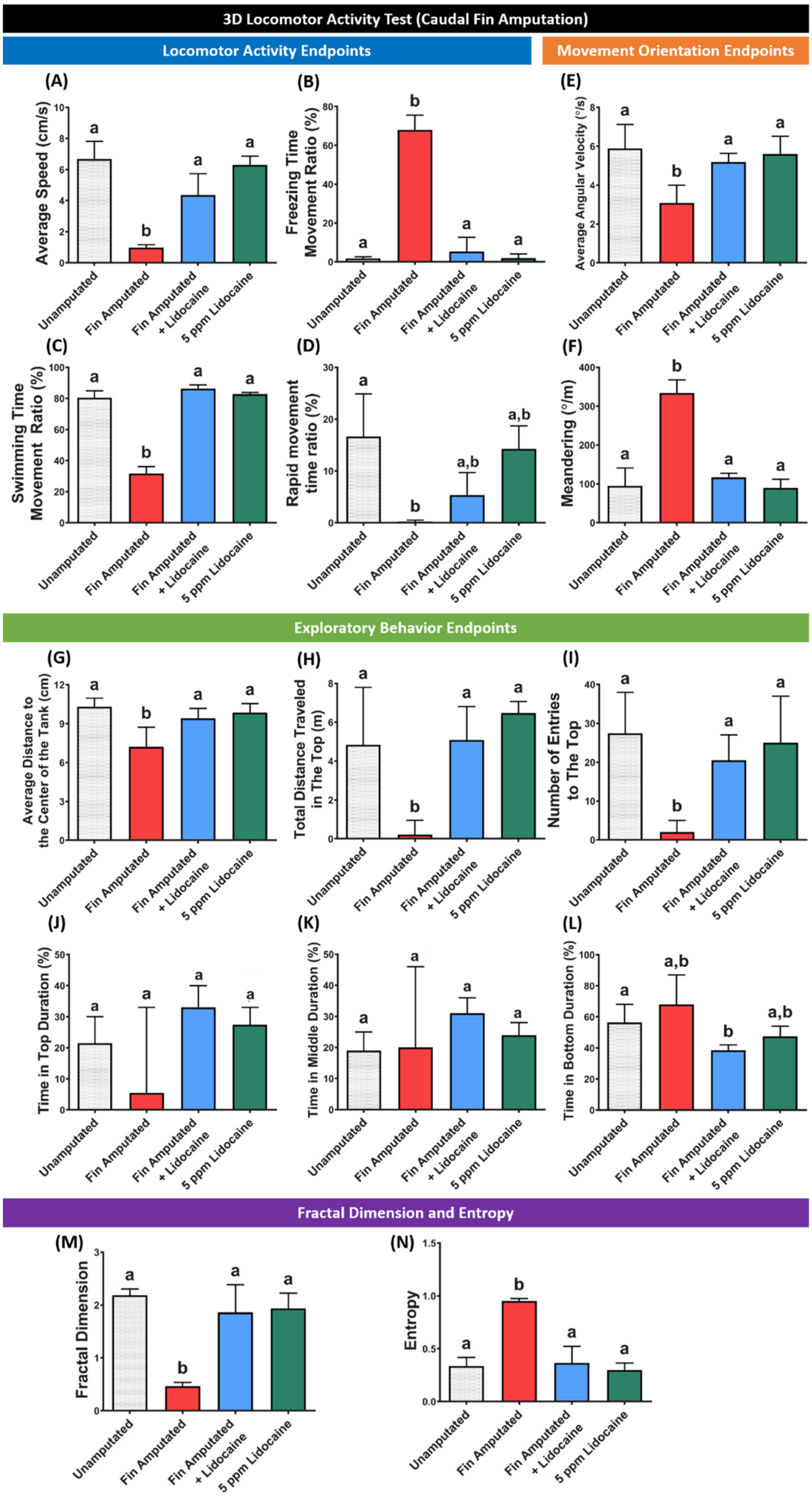

3D Locomotor Activity Test (Caudal Fin Amputation)
Locomotor Activity Endpoints
Movement Orientation Endpoints
(A)
Average Speed (cm/s)
(B)
Freezing Time Movement Ratio (%)
(E)
Average Angular Velocity (°/s)
(C)
Swimming Time Movement Ratio (%)
(D)
Rapid movement time ratio (%)
(F)
Meandering (°/m)
Exploratory Behavior Endpoints
(G)
Average Distance to the Center of the Tank (cm)
(H)
Total Distance Traveled in The Top (m)
(I)
Number of Entries to The Top
(J)
Time in Top Duration (%)
(K)
Time in Middle Duration (%)
(L)
Time in Bottom Duration (%)
Fractal Dimension and Entropy
(M)
Fractal Dimension
(N)
Entropy
Unamputated
Fin Amputated
Fin Amputated + Lidocaine
5 ppm Lidocaine
a
b
a,b

Figure S4. Comparison of behavior endpoints between unamputated, caudal fin-amputated, caudal fin-amputated with 5 ppm of lidocaine, and 5 ppm of lidocaine-treated zebrafish in the 3D locomotion test at 1-hour post-amputation (shoal size = six fish). The calculated **(A)** average speed, **(B)** freezing, **(C)** swimming, and **(D)** rapid movement time ratios indicated the fish locomotion while **(E)** average angular velocity and **(F)** meandering belong to the movement orientation endpoints group. Furthermore, to evaluate fish exploratory behaviors, **(G)** average distance to the center of the tank, **(H)** total distance traveled at the top, **(I)** number of entries to the top, time in the **(J)** top, **(K)** middle, and **(L)** bottom durations, were also calculated. Finally, **(M)** fractal dimension, **(N)** entropy, and **(O)** shoaling volume were also calculated. Data are expressed as median with 95% CI and were analyzed by Nested one-way ANOVA followed by Tukey's multiple comparisons test. Different letters signify a statistical difference ($P < 0.05$; $n = 3$ (18 fish)).
