# Supplementary material for: Fish 3D Clustering App (F3DCA): A Non-Supervised Clustering Method with a Machine Learning Autoencoder for Analyzing 3D Trajectories of Three Fish Species
Source: Biology (Basel). 2026 Oct 4;15(19):1768. doi: 10.3390/biology15191768 (PMC13664162; doi:10.3390/biology15191768)
Supplement: Supplementary file 1 [file biology-15-01768-s001.zip › Figure S6.pdf]

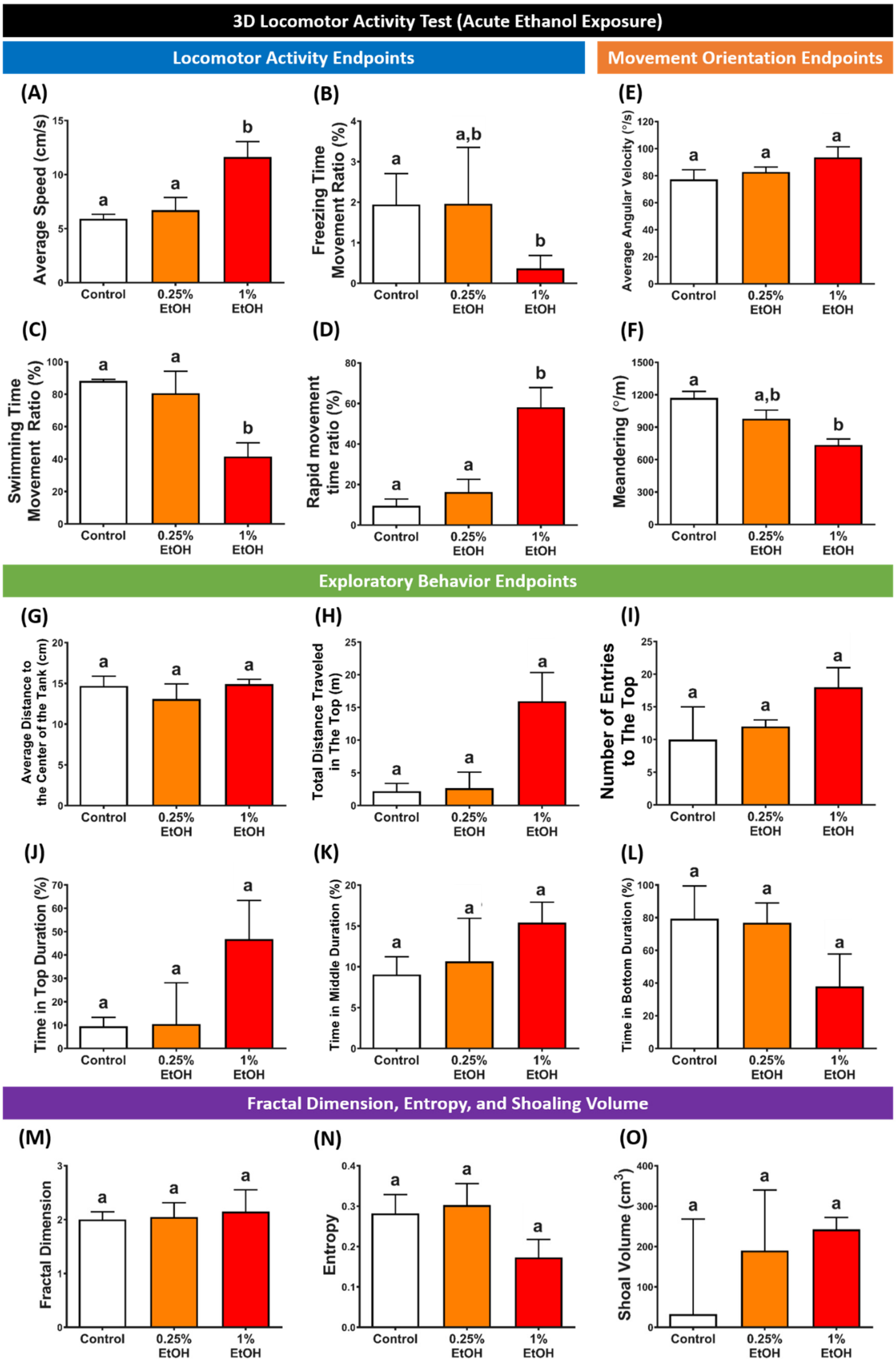

3D Locomotor Activity Test (Acute Ethanol Exposure)
Locomotor Activity Endpoints
Movement Orientation Endpoints
(A)
Average Speed (cm/s)
(B)
Freezing Time Movement Ratio (%)
(E)
Average Angular Velocity (°/s)
(C)
Swimming Time Movement Ratio (%)
(D)
Rapid movement time ratio (%)
(F)
Meandering (°/m)
Exploratory Behavior Endpoints
(G)
Average Distance to the Center of the Tank (cm)
(H)
Total Distance Traveled in The Top (m)
(I)
Number of Entries to The Top
(J)
Time in Top Duration (%)
(K)
Time in Middle Duration (%)
(L)
Time in Bottom Duration (%)
Fractal Dimension, Entropy, and Shoaling Volume
(M)
Fractal Dimension
(N)
Entropy
(O)
Shoal Volume (cm³)
Control
0.25% EtOH
1% EtOH
a
b
a,b

Figure S6. Comparison of behavior endpoints between zebrafish after acutely exposed with 0 (control), 0.25%, and 1% of ethanol in the 3D locomotion test at 1-hour post-amputation (shoal size = six fish). Control 18 fish = 3 shoals and each ethanol treatment 12 fish = 2 shoals. The calculated **(A)** average speed, **(B)** freezing, **(C)** swimming, and **(D)** rapid movement time ratios indicated the fish locomotion while **(E)** average angular velocity and **(F)** meandering belong to the movement orientation endpoints group. Furthermore, to evaluate fish exploratory behaviors, **(G)** average distance to the center of the tank, **(H)** total distance traveled at the top, **(I)** number of entries to the top, time in the **(J)** top, **(K)** middle, and **(L)** bottom durations, were also calculated. Finally, **(M)** fractal dimension, **(N)** entropy, and **(O)** shoaling volume were also calculated. Data are expressed as median with 95% CI and were analyzed by Nested one-way ANOVA followed by Tukey's multiple comparisons test, except (O), which was analyzed by Kruskal-Wallis test followed by Dunn's multiple comparisons test. Different letters signify a statistical difference($P < 0.05$; n control = 3 (18 fish), n EtOH treatment = 2 (12 fish)).
