# Supplementary material for: Fish 3D Clustering App (F3DCA): A Non-Supervised Clustering Method with a Machine Learning Autoencoder for Analyzing 3D Trajectories of Three Fish Species
Source: Biology (Basel). 2026 Oct 4;15(19):1768. doi: 10.3390/biology15191768 (PMC13664162; doi:10.3390/biology15191768)
Supplement: Supplementary file 1 [file biology-15-01768-s001.zip › Table S1.pdf]

Table S1. Summary of the fish behavioral endpoints measured in each experiment.

Index	Behavior	Behavior Endpoint
1-1	Locomotor Activity	Average Speed
1-2	Locomotor Activity	Freezing Movement Time Ratio
1-3	Locomotor Activity	Swimming Movement Time Ratio
1-4	Locomotor Activity	Rapid Movement Time Ratio
2-1	Movement Orientation	Average Angular Velocity
2-2	Movement Orientation	Meandering
3-1	Exploratory Behavior	Time in the Top Percentage
3-2	Exploratory Behavior	Time in Middle Percentage
3-3	Exploratory Behavior	Time in Bottom Percentage
3-4	Exploratory Behavior	Average Thigmotaxis
3-5	Exploratory Behavior	Total Distance Traveled in the Top
3-6	Exploratory Behavior	Total Entries to The Top
4	Fractal Dimension	Fractal Dimension
5	Entropy	Entropy
6	Shoaling Behavior	Shoaling Volume
